# Assessment of human plasma and urine sample preparation for reproducible and high-throughput UHPLC-MS clinical metabolic phenotyping†

**DOI:** 10.1039/d0an01319f

**Published:** 2020-07-29

**Authors:** Andrew D. Southam, Liam D. Haglington, Lukáš Najdekr, Andris Jankevics, Ralf J. M. Weber, Warwick B. Dunn

**Affiliations:** a School of Biosciences, University of Birmingham Edgbaston Birmingham B15 2TT UK a.d.southam@bham.ac.uk; b Phenome Centre Birmingham, University of Birmingham Edgbaston Birmingham B15 2TT UK; c Institute of Metabolism and Systems Research, University of Birmingham Edgbaston Birmingham B15 2TT UK

## Abstract

Clinical metabolic phenotyping employs metabolomics and lipidomics to detect and measure hundreds to thousands of metabolites and lipids within human samples. This approach aims to identify metabolite and lipid changes between phenotypes (*e.g.* disease status) that aid understanding of biochemical mechanisms driving the phenotype. Sample preparation is a critical step in clinical metabolic phenotyping: it must be reproducible and give a high extraction yield of metabolites and lipids, and in high-throughput studies it needs to be rapid. Here, we assessed the extraction of polar metabolites from human urine and polar metabolites and lipids from human plasma for analysis by ultra-high-performance liquid chromatography-mass spectrometry (UHPLC-MS) metabolomics and lipidomics. We evaluated several monophasic (urine and plasma) and biphasic (plasma) extractions, and we also tested alterations to (a) solvent–biofluid incubation time and temperature during monophasic extraction, and (b) phase partitioning time during biphasic extraction. Extracts were analysed by three UHPLC-MS assays: (i) hydrophilic interaction chromatography (HILIC) for urine and plasma, (ii) C_18_ aqueous reversed phase for urine, and (iii) C_18_ reversed phase for plasma lipids, and the yield and reproducibility of each method was assessed. We measured UHPLC-MS injection reproducibility as well as sample preparation reproducibility to assess sample solvent composition compatibility with UHPLC-MS and to pinpoint the origin of variance within the methods. For HILIC UHPLC-MS plasma and urine analysis, monophasic 50 : 50 methanol : acetonitrile had the most detected putatively-identified polar metabolites with high method reproducibility. This method had the highest lipid yield for plasma extracts analysed by the HILIC method. If lipid removal from the plasma polar HILIC extract is required, then the biphasic methanol/chloroform/water method is recommended. For C_18_ (aqueous) UHPLC-MS urine analysis, 50 : 50 methanol : water had high reproducibility and yield. For C_18_ UHPLC-MS plasma lipidomics, monophasic 100% isopropanol had the highest detection response of all annotated lipid classes with high reproducibility. Increasing monophasic incubation time and temperature had little benefit on metabolite and lipid yield and reproducibility for all methods.

## Abstract 2

In this study we assess multiple sample preparation methods for UHPLC-MS metabolic phenotyping analysis of human urine and plasma. All methods are discussed in terms of metabolite and lipid coverage and reproducibility.

## Graphical abstract

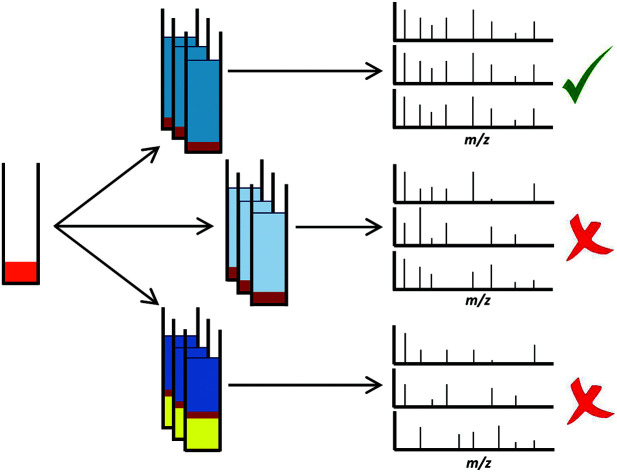

## 1. Introduction

Clinical metabolic phenotyping measures the relative amounts of a wide range of detectable small molecules (metabolites and lipids) in clinical samples (including plasma, serum, urine, primary and immortalised cells, tissue, cerebrospinal fluid, saliva and faeces^1,2^). This is commonly fulfilled by ultra-high performance liquid chromatography-mass spectrometry (UHPLC-MS),^3^ gas chromatography-mass spectrometry,^4^ direct infusion-mass spectrometry^5^ or nuclear magnetic resonance spectroscopy.^6^ Metabolic phenotyping can be rapid, high-throughput and a low-cost analysis^1^ (though not always applied with these goals), and is low- or non-invasive to patients when utilising samples such as plasma, serum or urine. The metabolome or lipidome (total metabolite or lipid measurement from samples) is a metabolic phenotype that defines interactions between genotype (or genotypes when considering human and gut microbe genomes), environment and lifestyle at a metabolic level.^1,2^ Levels of metabolites and lipids can be compared across phenotypes providing a powerful tool to rapidly understand and monitor many aspects of human health and disease including: disease biomarker identification and understanding disease mechanisms;^7^ patient stratification for disease classification or therapy (precision medicine);^8^ understanding drug toxicology;^9^ and understanding the benefits of exercise, nutrition and microbiome interaction.^10,11^

Sample preparation is a critical step in metabolomics and lipidomics. It needs to be reproducible, sensitive (to maximise metabolite and lipid extraction recovery) and should efficiently and rapidly denature proteins to halt residual enzymatic activity.^12–14^ Methods which are high-throughput and lend themselves to automation provide opportunities to increase sample-throughput and the size of studies. Here we assess and subsequently recommend appropriate protocols of plasma (and serum by its association) and urine preparation for analysis by UHPLC-MS metabolomics (both sample types) and lipidomics (plasma only). Plasma (or serum) is a whole organism extracellular matrix containing cumulative levels of metabolites and lipids: (a) from bodily ingestion or excretion and (b) taken up and excreted by organs and cells within the body. Plasma or serum preparation methods should remove other biochemicals (proteins, RNA and DNA) and extract metabolites and lipids into a liquid solvent suitable for analysis. Such approaches can be: (i) monophasic – the addition of a water-miscible organic solvent to extract soluble compounds into a single phase;^15^ and (ii) biphasic – the addition of immiscible organic solvent(s) and water (water provided by the biofluid and/or added water) to simultaneously extract hydrophilic metabolites and lipophilic metabolites into two separate phases.^13^ Monophasic extracts can be immediately analysed (post-centrifugation) if the extraction solvents are compatible with UHPLC-MS.^12,15^ For biphasic extractions^13^ (and monophasic extractions using non-UHPLC-MS-compatible solvents^16^) samples are dried and reconstituted in an appropriate solvent before analysis, thus increasing sample preparation complexity and time. The extraction solvent(s) influences metabolite/lipid solubility and protein removal efficiency^12,15^ (sample stability is decreased if enzyme activity is not fully inactivated^14^).

For monophasic polar metabolite extraction from serum or plasma, methanol is highly regarded, giving good metabolite extraction yield, efficient deprotenisation and high reproducibility.^15,17,18^ Ethanol : methanol (1 : 1) has shown positive results,^18^ but decreasing solvent polarity would limit highly polar compound recovery. Acetonitrile divides opinion: it has been shown as both good^19^ and bad^17,20^ in terms of reproducibility, extraction efficiency and protein denaturation. Monophasic serum or plasma lipid extraction solvents compatible with direct UHPLC-MS analysis include isopropanol, 1-butanol : methanol mix, methanol, ethanol and acetonitrile.^12,20,21^ Monophasic lipid extraction solvents that require drying and reconstitution before analysis include methanol : chloroform^21,22^ and methanol : methyl *tert*-butyl ether (MTBE) : chloroform.^16,22^ Isopropanol is highly regarded for lipid recovery and method simplicity.^12^

Biphasic plasma and serum extractions simultaneously extract polar compounds and lipids into two separate phases. This is beneficial (i) where insufficient sample volume exists for two separate extractions, (ii) to remove salts and polar compounds from the lipid extract,^23^ and (iii) to remove glycerophospholipids from the polar extract which can cause matrix effects. The latter two points [(ii) and (iii)] reduce UHPLC-MS ionisation suppression^24^ and MS source fouling, which potentially can increase detection sensitivity. Biphasic extractions are longer protocols with more steps than monophasic preparations and often (but not always) extracts need drying and reconstitution before UHPLC-MS analysis. This reduces extraction speed and potentially increases technical variance. Chloroform/methanol/water,^12,13,16,22^ dichloromethane/methanol/water,^12^ dichloromethane/methanol/saline^25^ and MTBE/methanol/water^12,13,16^ have been applied. Dichloromethane/methanol/saline was comparable to chloroform/methanol/saline for serum lipid extraction efficiency,^25^ while MTBE/methanol/water plasma extraction using an optimised ratio of 2.6 : 2.0 : 2.4 was more reproducible than chloroform/methanol/water (for lipid extracts) and had a higher polar metabolite yield.^13^ Biphasic serum and plasma extractions generally have lower polar metabolite^26^ and lipid^12,16,22^ yields compared to monophasic extractions.

Urine is a whole organism excretion matrix, which informs on whole body changes including gut microbial metabolism. Urine from patients with healthy kidneys contain very little protein, therefore the use of protein-denaturing organic solvents is not essential.^27^ Preparation protocols include: (i) urine dilution with water and centrifugation prior to UHPLC-MS analysis^28^ (using sodium azide^28^ or the sample filtering^29^ to prevent microorganism growth); and (ii) the addition of organic solvents including methanol or acetonitrile.^30,31^ Organic solvent use is beneficial for clinical metabolic phenotyping as it rapidly eliminates microorganisms and removes any residual protein from samples, which is important if patients have renal damage induced proteinuria – a side effect of some diseases (*e.g.* multiple myeloma^32^) and clinical treatments (*e.g.* chemotherapy^33^).

An important consideration for monophasic extraction methods is the time and temperature used during the incubation of biofluids with solvent to achieve the most efficient metabolite and lipid extraction. However, the limited available evidence suggests these factors are likely not important, *e.g.* varying time (10 or 120 min) and temperature (ice or −20 °C) during the monophasic extraction of plasma with methanol had little effect on peak areas.^17^ For biphasic extractions of tissue samples, increasing phase separation time can increase compounds reproducibility and yield.^14^

Several biofluid preparation studies exist; however, studies often have different conclusions which are sometimes conflicting.^17,19^ Published work rarely investigates lipids extracted by polar preparation methods (with few lipid species covered in the studies that exist^34^). Previous studies do not indicate the proportion of variance arising from (a) the sample preparation and (b) the detection method, which is key to understand where in the whole process improvements could be made to improve the methods. Such analysis would also allow an understanding of the compatibility of different sample solvent composition with UHPLC-MS methods. Few studies test changes in variance and yield in relation to key incubation preparation steps: we were only able to find a single study that tests changes to solvent–biofluid incubation^17^ and none that test the influence of phase partition incubation on yield and reproducibility for biofluid biphasic methods. In this current study we provide greater clarity on the above points. Here, we characterise several different solvents for the monophasic and biphasic extraction of polar metabolites and lipids from plasma, and the monophasic extraction of polar metabolites from urine. We test the influence of (i) monophasic solvent–biofluid incubation time and temperature and (ii) biphasic partition time. Methods were compared in terms of reproducibility (peak area relative standard deviation) and compound recovery (number and response of detected identified compounds – putative and MS/MS annotated). A key aim was to test methods that avoid sample drying and re-constitution (monophasic preparations where samples are directly analysed by UHPLC-MS after preparation). Thus, we also test solvent suitability for UHPLC-MS by assessing injection replicates. For polar sample preparations we also investigate the relative amounts and range of lipids that were extracted by each method.

## 2. Experimental section

### 2.1. Materials

Human urine and plasma (Seralab, BioIVT; received frozen) were split into 50 μL aliquots and frozen at −80 °C until preparation. Acetonitrile (ACN), methanol (MeOH), water (H_2_O), and isopropanol (propan-2-ol, IPA) were all LC-MS grade (Optima brand, Fisher Scientific). Chloroform (CHCl_3_), dichloromethane (DCM), and methyl *tert*-butyl ether (MTBE) were all HPLC grade (Fisher Scientific).

### 2.2. Experimental design

For each tested sample preparation condition: (i) *n* = 3 sample preparation replicates were analysed, and (ii) *n* = 3 UHPLC-MS injection replicates were analysed (a single preparation re-injected 3 times). Data was assessed: (i) across sample preparation replicates to define sample preparation variance and yield, and (ii) across UHPLC-MS injection replicates to define UHPLC-MS variance and yield.

### 2.3. Urine monophasic sample preparation

50 μL urine (thawed on ice) and 150 μL of ice-cold solvent (100% ACN or 100% MeOH or 50 : 50 ACN : H_2_O or 50 : 50 MeOH : H_2_O or 50 : 50 ACN : MeOH) were mixed, vortexed (120 s) and then: (i) not incubated, (ii) incubated at 4 °C for 60 min or (iii) incubated at −20 °C for 60 min. Samples were centrifuged (21 000*g*, 20 min, 4 °C) and 100 μL of the supernatant was aliquoted into a low recovery volume HPLC vial (Chromatography Direct, UK). *n* = 4 extraction replicates were conducted for each solvent type and preparation condition (*n* = 3 of these were analysed; and the remaining *n* = 1 was used to create a pooled QC sample, see ‘*Extract blank and quality control (QC) sample preparation*’ section).

### 2.4. Plasma monophasic sample preparation

This protocol was the same as the urine monophasic preparation except 50 μL plasma was used instead of urine and different solvents were used: (a) for polar metabolite extraction and subsequent HILIC UHPLC-MS analysis the added solvent was 100% ACN or 100% MeOH or 50 : 50 ACN : MeOH; and (b) for lipid extraction and subsequent C_18_ reversed-phase lipid UHPLC-MS analysis the added solvent was 100% ACN or 25 : 75 ACN : IPA or 50 : 50 ACN : IPA or 75 : 25 ACN : IPA or 100% IPA. In all cases the biofluid : solvent ratio was 1 : 3.

### 2.5. Plasma biphasic sample preparation

Three biphasic extractions were applied (a) CHCl_3_ : MeOH : H_2_O, (b) DCM : MeOH : H_2_O or (c) MTBE : MeOH : H_2_O, as described previously.^13,14,23^ For the CHCl_3_ : MeOH : H_2_O method, 320 μL ice-cold methanol and 78 μL ice-cold water was added to plasma (50 μL, thawed on ice) and vortexted (60 s). Then, 320 μL ice-cold chloroform and 160 μL ice-cold water was added and sample was vortexed (60 s), incubated (on ice, 10 min) and centrifuged (21 000*g*, 10 min, 4 °C). The final solvent ratio was 2 : 2 : 1.8 CHCl_3_ : MeOH : H_2_O (assuming plasma is 100% water). The final biofluid : solvent ratio was 1 : 17.6 for this method. The sample was set at room temperature (approximately 20 °C) to allow biphase partitioning for either: (i) 1 min or (ii) 20 min 70% of the polar phase (377 μL) was removed and dried in a SpeedVac sample concentrator (Savant SPD111V230, Thermo Fisher Scientific). 70% of the non-polar phase (273 μL) was removed and dried using a nitrogen blow down drier (Techne FSC400D, Thermo Fisher Scientific). The DCM : MeOH : H_2_O method was conducted as above except: dichloromethane was used instead of chloroform; here 70% of the final polar phase was 442 μL and 70% of the non-polar phase was 228 μL. The final biofluid : solvent ratio was 1 : 17.6 for this method. The MTBE : MeOH : H_2_O method used different solvent ratios to the two methods above (final solvent ratio of 2.6 : 2 : 2.4 MTBE : MeOH : H_2_O; final biofluid : solvent ratio was 1 : 21.4).^13^ This was conducted as for the CHCl_3_ : MeOH : H_2_O method above except: initially 320 μL ice-cold methanol and 78 μL ice-cold water was added to the plasma (50 μL); and the second solvent addition step was 416 μL ice-cold MTBE and 256 μL ice-cold water. Here, 70% of the final polar phase was 549 μL and 70% of the non-polar phase was 235 μL. All polar extracts were resuspended in 140 μL 75 : 25 ACN : H_2_O and non-polar extracts were resuspended in 140 μL 75 : 25 IPA : H_2_O, to match the plasma dilution of the monophasic methods (and the same solvent composition as the 100% ACN or 100% IPA monophasic preparations, above). Samples were vortexed (30 s), centrifuged (21 000*g*, 20 min, 4 °C) and 100 μL of the supernatant was aliquoted into a low recovery volume HPLC vial. *n* = 4 extraction replicates were done for each solvent type and extraction condition (*n* = 3 of these were analysed; and the remaining *n* = 1 was used to create a pooled QC sample, see ‘*Extract blank and quality control (QC) sample preparation*’ section).

### 2.6. Extract blank and quality control (QC) sample preparation

Extract blank samples were prepared for each method by substituting 50 μL of water for the plasma or urine. A pooled QC sample was created for each UHPLC-MS assay by pooling the 4^th^ extraction replicate prior to the final centrifugation step for each method. The QC pool was vortexed (60 s), centrifuged (21 000*g*, 10 min, 4 °C) and 100 μL of the supernatant was aliquoted into low recovery volume HPLC vials.

### 2.7. Ultra-high performance liquid chromatography-mass spectrometry (UHPLC-MS)

The samples (maintained at 4 °C) were analysed by applying three Ultra Performance Liquid Chromatography-Mass Spectrometry (UHPLC-MS) methods using a Dionex UltiMate 3000 Rapid Separation UHPLC system (Thermo Fisher Scientific, MA, USA) coupled with a heated electrospray Q Exactive Focus mass spectrometer (Thermo Fisher Scientific, MA, USA). Three separate UHPLC-MS assays were applied (assay 1, assay 2, and assay 3). Urine preparations were analysed by assays 1 and 2. Polar plasma preparations were analysed by assay 1. Lipid (non-polar) plasma preparations were analysed by assay 3.

Assay 1 was a HILIC method using an Accucore-150-Amide-HILIC column (100 × 2.1 mm, 2.6 μm, Thermo Fisher Scientific, MA, USA) as used previously.^35^ Mobile phase A: 95% acetonitrile/water (10 mM ammonium formate, 0.1% formic acid); mobile phase B: 50% acetonitrile/water (10 mM ammonium formate, 0.1% formic acid). Gradient: *t* = 0.0, 1% B; *t* = 1.0, 1% B; *t* = 3.0, 15% B; *t* = 6.0, 50% B; *t* = 9.0, 95% B; *t* = 10.0, 95% B; *t* = 10.5, 1% B; *t* = 14.0, 1% B. All changes were linear (curve = 5) and the flow rate was 0.50 mL min^−1^. Column temperature was 35 °C and injection volume was 2 μL. Data were acquired in positive and negative ionisation modes separately (70–1050 *m*/*z*) with a resolution of 70 000 (FWHM at *m*/*z* 200). Ion source parameters: sheath gas = 53 arbitrary units, aux. gas = 14 arbitrary units, sweep gas = 3 arbitrary units, spray voltage = 3.5 kV (positive ion)/2.7 kV (negative ion), capillary temp. = 269 °C (positive ion)/320 °C (negative ion), aux. gas heater temp. = 438 °C (positive ion)/320 °C (negative ion). Data dependent MS2 in ‘Discovery mode’ was applied to three QC samples over three mass ranges (70–200 *m*/*z*; 200–400 *m*/*z*; 400–1000 *m*/*z*) using following settings: resolution = 17 500; isolation width = 3.0 *m*/*z*; stepped normalised collision energies = 25, 60, 100%.

Assay 2 was an aqueous reversed phase method using a Hypersil GOLD C_18_ (aq.) column (100 × 2.1 mm, 1.9 μm; Thermo Fisher Scientific, MA, USA). Mobile phase A: water (0.1% formic acid); mobile phase B: acetonitrile (0.1% formic acid). Gradient: *t* = 0.0, 1% B; *t* = 0.5, 1% B; *t* = 2.0, 50% B; *t* = 9.0, 99% B; *t* = 10.0, 99% B; *t* = 10.5, 1% B; *t* = 15.0, 1% B. All changes were linear (curve = 5) and the flow rate was 0.30 mL min^−1^. Column temperature was 45 °C and injection volume was 2 μL. Data were acquired in positive and negative ionisation modes separately (100–1500 *m*/*z*) with a resolution of 70 000. Ion source parameters: sheath gas = 48 arbitrary units, aux. gas = 11 arbitrary units, sweep gas = 2 arbitrary units, spray voltage = 3.5 kV (positive ion)/2.5 kV (negative ion), capillary temp. = 256 °C, aux. gas heater temp. = 413 °C. Data dependent MS2 in ‘Discovery mode’ was applied to three QC samples over three mass ranges (100–300 *m*/*z*; 300–600 *m*/*z*; 600–1500 *m*/*z*) using following settings: resolution = 17 500; isolation width = 3.0 *m*/*z*; stepped normalised collision energies = 20, 50, 80%.

Assay 3 was a reversed phase lipid analysis method using a Hypersil GOLD C_18_ column (100 × 2.1 mm, 1.9 μm; Thermo Fisher Scientific, MA, USA) as used previously.^35^ Mobile phase A: 60% acetonitrile/water (10 mM ammonium formate, 0.1% formic acid); mobile phase B: 85.5% propan-2-ol/9.5% acetonitrile/5% water (10 mM ammonium formate, 0.1% formic acid). Gradient: *t* = 0.0, 20% B; *t* = 0.5, 20% B, *t* = 8.5, 100% B; *t* = 9.5, 100% B; *t* = 11.5, 20% B; *t* = 14.0, 20% B. All changes were linear (curve = 5) and the flow rate was 0.40 mL min^−1^. Column temperature was 55 °C and injection volume was 2 μL. Data were acquired in positive and negative ionisation mode separately (150–2000 *m*/*z*) with a resolution 70 000. Ion source parameters: sheath gas = 50 arbitrary units, aux. gas = 13 arbitrary units, sweep gas = 3 arbitrary units, spray voltage = 3.5 kV (positive ion)/2.5 kV (negative ion), capillary temp. = 263 °C, aux. gas heater temp. = 425 °C. Data dependent MS2 in ‘Discovery mode’ was applied to three QCs over three mass ranges (200–400 *m*/*z*; 400–700 *m*/*z*; 700–1500 *m*/*z*) using the following settings: resolution = 17 500; isolation width = 3.0 *m*/*z*; stepped normalised collision energies = 20, 50, 80%.

Thermo ExactiveTune (2.8 SP1, build 2806) software controlled the instrument. All data were acquired in profile mode. Quality control (QC) samples were analysed as the first ten injections and then every tenth injection with two at the end of the analytical batch. An extract blank sample was analysed as the 6th injection. Extract blank samples from all extraction methods were analysed at the end of the batch.

### 2.8. Raw data processing and metabolite annotation

Fig. S1† visualises the data processing pipeline. Vendor format raw data files (.RAW) were converted to the mzML file format using ProteoWizard software (settings: peakPicking at vendor msLevel = 1; selected options = 64-bit, write index, use zlib compression, TPP compatible).^36^ Deconvolution was performed by XCMS software^37^ (version 1.46.0 running in the Galaxy environment^38^) applying min peak width (urine reversed phase = 3; HILIC = 4; lipids = 6); max. peak width (30); ppm (urine reversed phase = 11; HILIC = 12; lipids = 14); mzdiff (0.001); bw (urine reversed phase = 0.5; HILIC and lipids = 0.25); mzwid (0.01); minfrac (0.2). A data matrix of peak areas for metabolite features (*m*/*z*-retention time pairs) *vs.* samples were constructed. Putative metabolite annotation was performed applying PUTMEDID-LCMS (5 ppm mass error for metabolite matching; 2 s retention time range for feature grouping).^39^ Multiple annotations (*e.g.* isomeric compounds) could be observed for a single detected metabolite feature. Throughout this article, the term putatively annotated metabolite refers to either single metabolites or groups of molecules with the same retention time and the same accurate *m*/*z*.

To generate more robust compound annotations, QC sample UHPLC-MS/MS data were matched to MS/MS databases using either LipidSearch software (lipid annotation; version 4.2.18, Thermo Fisher Scientific) or Compound Discoverer 3 software (polar compound and some lipid annotations; version 3, Thermo Fisher Scientific) (Fig. S1†). Lipid features within the UHPLC-MS/MS data were searched against the entire *in silico* HCD MS/MS database (5 ppm mass error). Only annotations graded A-C were used for annotation purposes (Grade A – all fatty acyl chains and class were completely identified; Grade B – some fatty acyl chains and the class were identified; Grade C – either the lipid class or some fatty acyls were identified). MS/MS annotations using Compound Discoverer were graded by HighChem HighRes algorithm within the Compound Discoverer software (annotations with a score >60 were retained for identification purposes). LipidSearch and Compound Discoverer annotations were aligned to the XCMS outputs using the R programming language (https://www.R-project.org), using 5 ppm mass error and 20 s retention time tolerance window.

### 2.9. Data filtering using QC samples to generate the putative metabolite and lipid data shown throughout the study

An overview of data filtering is shown in Fig. S1.† The first 8 QC samples and QC samples with a total peak area (of all metabolites) exceeding ±25% of the median QC total peak area were removed from the data matrix. Features were retained in the data matrix if they were: present in >70% of QC samples; had a peak area relative standard deviation (RSD) < 30% across QC samples; and had an extract blank/mean QC area ratio of <5%. This filtering retains highly robust and reproducible features across 8–14 QC injections over a >24 h period, an important step as feature stability and reproducibility over a long time period is essential for metabolic phenotyping studies. Only features with putative metabolite annotations (a matched metabolic formula from PUTMEDID-LCMS) were retained. For each putatively annotated metabolite, only the metabolite ion form with the largest peak area were retained (using mean peak area of all samples across all preparation conditions).

### 2.10. Statistics

For each preparation method, un-normalised peak area relative standard deviation (RSD) values were calculated for peaks present in all three sample preparation replicates or UHPLC-MS injection triplicates. Venn diagrams^40^ were generated using the R programming language (https://www.R-project.org). For principal components analysis (PCA) the above filtered data matrices were processed to retain peaks present across >80% of all samples. Then matrices were normalised (probabilistic quotient normalisation^41^), missing data values were imputed (*k* nearest neighbour, kNN) and a generalised log transformation was applied.^42^ PCA was conducted using PLS Toolbox in Matlab. The other processing steps were executed using the R/Bioconductor packages pmp (https://bioconductor.org/packages/release/bioc/html/pmp.html) and structToolbox (https://bioconductor.org/packages/release/bioc/html/structToolbox.html).

## 3. Results and discussion

### 3.1. Optimised urine sample preparation for the C_18_ aqueous reversed-phase UHPLC-MS assay

**Fig. 1:**
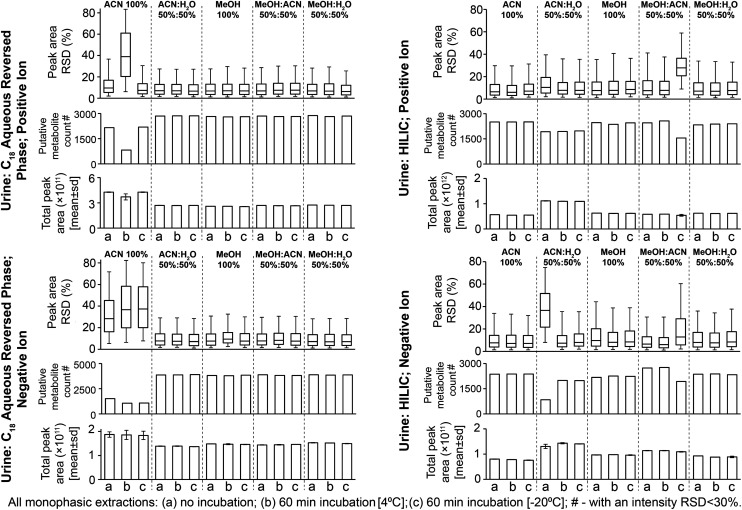
Sample preparation replicate data for putatively annotated metabolites from all urine sample preparation methods. Data were filtered using QC samples and then putatively annotated (Experimental section). Relative standard deviations (RSD; shown as interquartile ranges and error bars as the 95^th^ percentile) are calculated on peak areas where the putative metabolite is present in all three sample preparation replicates. Putatively annotated metabolite counts are those in three sample preparation replicates with a peak area RSD < 30%.

Four of the five methods (50 : 50 ACN : H_2_O, 50 : 50 MeOH : H_2_O, 100% MeOH, 50 : 50 ACN : MeOH) had high reproducibility for positive and negative ion modes: median sample preparation peak area relative standard deviations (RSDs) were 6.7–7.7% (Fig. 1, no solvent incubation) and median UHPLC-MS injection peak area RSDs were 6.5–7.3% (Fig. S2,† no solvent incubation). Preparation replicates for these methods were tightly clustered on the principal components analysis (PCA) scores plot (Fig. S3†). 100% ACN had poor sample preparation and injection reproducibility (Fig. 1 and S2†) and decreased putative metabolite counts relative to other methods. This effect was less apparent in the positive ion data compared to the negative ion data (Fig. 1). In the positive ion data only 100% ACN preparation with 60 min incubation at 4 °C had increased variability and low reproducible putative metabolite counts, which was attributable to the single outlier visible on the PCA scores plot (Fig. S3;† post outlier removal median RSD was 5.2% and the count of putative metabolites with an RSD < 30% was 2269). In this instance, the data suggests that it was either a single miss injection or that this method has sporadic poor reproducibility. In negative ion, the poor reproducibility was also apparent in the UHPLC-MS injection data (Fig. S2†). Observing the UHPLC-MS injection data, situations where the reproducibility was poor was associated with large spectral variances of peaks with <1 minute retention time (Fig. S4†). Further investigation revealed that features putatively annotated as citrate and isocitrate were present in this early retention time window and showed high peak area variability in the negative ion data (Fig. S4†). Given that these compounds would predominantly ionise negatively, this could indicate why negative ion mode was generally lower reproducibility than positive ion mode. In support of this observation, inspection of MS/MS annotated peaks extracted by 100% ACN showed that several other acidic compounds have poor reproducibility in the negative ion data (Table S1†). In 100% ACN the peaks with a retention time of <1 minute had higher peak areas than in the other preparation methods (Fig. S4 and S5†), accounting for the general higher total peak area in 100% ACN compared to other methods. We conclude that 100% ACN is not recommended for this method based on poor reproducibility. Potential reasons for poor reproducibility include immiscibility either (i) within the final prepared sample or (ii) when the sample [high ACN content] mixes with the initial mobile phase conditions [high water content], which results in sporadic injection variance. All other methods did not experience this issue (Fig. 1 and Fig. S2†).

**Fig. 2:**
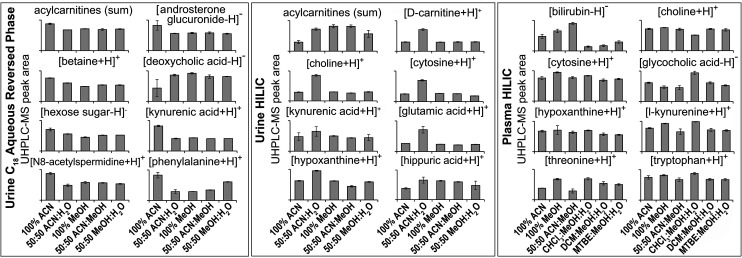
Selected MS/MS-annotated metabolites detected by HILIC and C_18_ aqueous reversed phase analysis of polar metabolite preparations of plasma or urine. UHPLC-MS peak area for each annotated compound is shown as mean ± sd (*n* = 3 preparation replicates; no solvent incubation for monophasic and 1 min partition time for biphasic methods). Metabolites were annotated from QC UHPLC-MS/MS data using Compound Discoverer and Lipid Search software (*m*/*z*, retention times, MS/MS annotation grade, mean peak area and standard deviations for each compound shown here plus additional compounds are shown in Table S1†). All annotated acylcarnitines were summed.

The four reproducible methods (*i.e.* excluding 100% ACN) had similar numbers of detected putative metabolites (Fig. 1; 74–78% putative metabolites were detected by all four methods, Fig. S6†). The UHPLC-MS chromatograms and many classes of metabolite showed similar detection responses across these methods (Fig. 2, Fig. S5 and Table S1†). These findings demonstrate much overlap between the four methods, however, the PCA scores plot highlights small differences in metabolite responses or detection between methods (Fig. S3†). Varying urine–solvent incubation time or temperature had minimal effect on reproducibility, putative metabolite count, metabolite response, or metabolic profile (Fig. 1, Fig. S3 and Table S2†) suggesting incubations have minimal effect on sample extraction (extraction being the removal of any residual protein present in urine from renal damage or bacterial infection). The biofluid–solvent incubation data can also be used to assess the stability of the sample post-extraction (given that protein content of normal urine samples is expected to be close to zero). For all urine sample preparation methods analysed by the C_18_ aqueous reversed-phase UHPLC-MS assay, extremely few features showed significant change after incubation with solvent (for all methods, <0.5% features were statistically altered with incubation, FDR < 5%; Table S3†). This demonstrated the high stability of all sample preparation methods post-preparation. We conclude that any tested method except 100% ACN is suitable for C_18_ aqueous reversed phase UHPLC-MS urine metabolomics and urine–solvent incubation is not required. We recommend 50 : 50 MeOH : H_2_O as it avoids acetonitrile use which may mix poorly with water at the beginning of our C_18_ aqueous reversed phase UHPLC-MS assay.

### 3.2. Optimised urine sample preparation for the HILIC UHPLC-MS assay

All methods had median sample preparation and UHPLC-MS injection peak area RSDs of <10% (except 50 : 50 ACN : H_2_O with no solvent incubation and 50 : 50 MeOH : ACN with 60 min −20 °C incubation; Fig. 1 and S2†), demonstrating little sample preparation or injection technical variance. PCA scores plots further demonstrate high sample preparation reproducibility (Fig. S7†). The low sample preparation and UHPLC-MS injection reproducibility of 50 : 50 ACN : H_2_O (no incubation, negative ion; Fig. 1 and S2†) suggested poor injection reproducibility as seen for 100% ACN method with the urine C_18_ aqueous reversed phase assay (section 3.1). Few MS/MS annotated compounds were observed in the negative ion HILIC assay, however MS/MS-annotated cholic acid had poor reproducibility here (Table S1†). Reproducibility was improved by urine–solvent incubation, however given that the reproducibility issues may be caused by immiscibility of the sample with initial mobile phase conditions (potentially an issue with high water mixing with high ACN at the beginning of an analysis as suggested for the urine C_18_ aqueous reversed phase assay, section 3.1) this method is not recommended. Poor reproducibility of 50 : 50 MeOH : ACN (60 min incubation, −20 °C; Fig. 1) was caused by low total peak area of one sample preparation replicate. This suggested a detrimental effect of incubation; however, the metabolic profile was corrected by normalisation as viewed on the PCA scores plot (Fig. S7†).

The four reproducible methods (100% ACN, 50 : 50 MeOH : H_2_O, 100% MeOH, 50 : 50 ACN : MeOH) had similar putative metabolite counts in positive ion mode (Fig. 1): 100% ACN (2505 putatively identified metabolites), 100% MeOH (2468), 50 : 50 ACN : MeOH (2452) and 50 : 50 MeOH : H_2_O (2339). In negative ion mode, 50 : 50 ACN : MeOH had the highest putative metabolite count (2733) followed by 100% ACN (2369), MeOH : H_2_O (2369) and MeOH (2173) (Fig. 1). There was overlap between methods (62–67% [positive ion] and 52–66% [negative ion] of putative metabolites were detected by all four methods (Fig. S8†), however this was less overlap than for the urine C_18_ aqueous reversed phase UHPLC-MS assay (Fig. S6†). This is partly due to lower UHPLC-MS injection reproducibility of the urine HILIC assay compared to the urine C_18_ aqueous assay (Fig. S2†), which negatively affects putative metabolite counts with an RSD threshold. Consistent with this, 50 : 50 ACN : MeOH (HILIC negative ion) had the lowest RSD and highest putative metabolite count (RSD < 30%), demonstrating the importance of method reproducibility. UHPLC-MS chromatograms (Fig. S9†) and metabolite responses of the four reproducible methods were similar (Fig. 2). However, some differences between methods were apparent on the PCA scores plot (Fig. S7†). This was partly due to the acylcarnitine response (highest in 100% MeOH and 50 : 50 ACN : MeOH, and lowest in 100% ACN; Fig. 2), suggesting ACN is less effective for lipid recovery than MeOH. The 50 : 50 ACN : H_2_O had lower putative metabolite counts but higher total peak area than all other methods. This appears to be due to 50 : 50 ACN : H_2_O providing the best environment for very polar metabolites (in terms of solubility and/or ionisation efficiency, Fig. 2 and Table S1†). However, this method performs much poorer with less polar compounds, which is highlighted by high detection responses of very polar acylcarnitines but low detection responses of less polar acylcarnitines (Fig. S10†), which could account for the lower putative metabolite counts.

Varying the urine–solvent incubation time or temperature had little effect on reproducibility, putative metabolite count, metabolite response, or metabolic profile (Fig. 1, Fig. S7 and Table S2†). For all urine sample preparation methods analysed by the HILIC UHPLC-MS assay, extremely few features showed significant change after incubation with solvent (for all methods, <0.6% features were statistically altered with incubation, FDR < 5%; Table S3†). This is consistent with the C_18_ aqueous reversed-phase UHPLC-MS data and demonstrates the high stability of all sample preparation methods post-preparation. We conclude that, in terms of reproducibility and detected putative metabolites, 50 : 50 ACN : MeOH with no urine–solvent incubation is recommended for both positive and negative ionisation HILIC analysis. Although 50 : 50 ACN : H_2_O appeared to provide a greater detection response for the eight chosen metabolites (Fig. 2), the reproducibility of replicate injections and the number of putative metabolites detected was not analytically appropriate.

### 3.3. Optimised plasma sample preparation for the HILIC UHPLC-MS assay

**Fig. 3:**
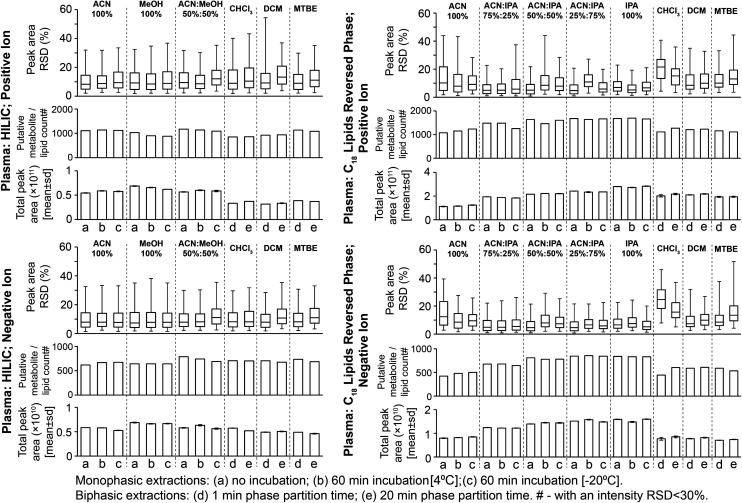
Sample preparation replicate data for putatively annotated metabolites and lipids from all plasma sample preparation methods. This data was filtered using QC samples and then putatively annotated (Methods section). Relative standard deviations (RSD; shown as interquartile ranges and error bars as the 95^th^ percentile) are calculated on peak intensities where the putative metabolite/lipid is present in all three sample preparation replicates. Putatively annotated metabolite/lipid counts are those present in three sample preparation replicates with an area RSD < 30%.

**Fig. 4:**
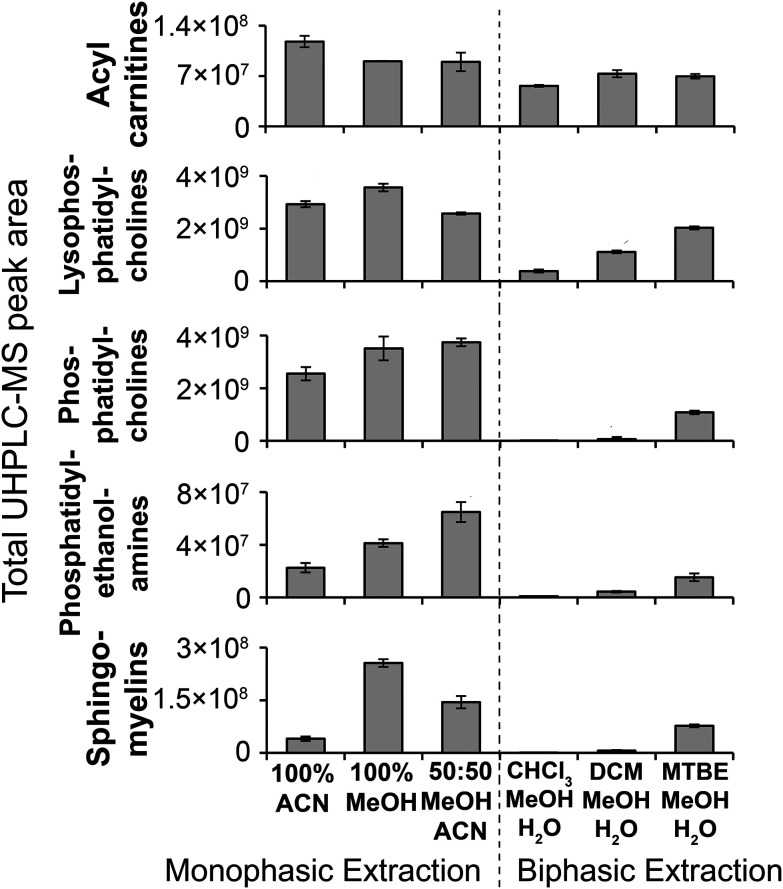
MS/MS-annotated lipids detected in the HILIC UHPLC-MS analysis of polar metabolite extractions of plasma. UHPLC-MS positive ion total peak area for each annotated lipid class is shown as mean ± sd (*n* = 3 preparation replicates; no solvent incubation for monophasic and 1 min partition time for biphasic methods). Lipids were annotated from QC UHPLC-MS/MS data using Lipid Search software (*m*/*z*, retention times, MS/MS annotation grade, mean peak area and standard deviations for each lipid species is shown in Table S1†).

Considering monophasic preparation methods, median sample preparation peak area RSDs (no incubation) were 8.2–9.4% and 7.3–8.0% for positive and negative ion, respectively (Fig. 3) and median UHPLC-MS injection peak area RSDs were 5.9–8.1% and 6.5–7.0% for positive and negative ion, respectively (Fig. S11†). Along with the tight clustering of sample preparation replicates on the PCA scores plot (Fig. S12†), this demonstrates very little sample preparation and injection technical variance. The monophasic method (no solvent incubation) with the highest number of reproducible putative metabolites was 50 : 50 ACN : MeOH for both positive (1173) and negative (790) ion modes (Fig. 3), followed by 100% ACN (positive 1111; negative 620) and 100% MeOH (positive 1032; negative 642). The UHPLC-MS chromatograms and metabolites showed similar detection responses across the monophasic methods (Fig. 2, Fig. S13 and Table S1†). There was an apparent trend for amino acids and related compounds (kynurenine, tryptophan, threonine) where the response was highest in 100% MeOH, indicating that methanol is the best for extraction of such compounds or can provide the greatest ionisation efficiency (Fig. 2 and Table S1†). Multiple lipid classes were extracted by the monophasic methods, with 50 : 50 MeOH : ACN and 100% MeOH methods having a higher detection response than 100% ACN (for phosphatidylcholines, phosphatidylethanolamines and sphingomyelines; Fig. 4). Solvent incubation had minimal effect on sample preparation reproducibility or the number of detected putative metabolites (Fig. 3), however solvent incubation during the 50 : 50 ACN : MeOH method (60 min at 4 °C or −20 °C) induced a shift on the PCA scores plot (Fig. S12†). Several phosphatidylcholine, phosphatidylinositol and sphingomyelin species had higher responses after incubation (Table S2†), indicating enhanced lipid extraction with incubation for this method. The 100% ACN method also showed higher response of some lipids after solvent incubation (Table S2†).

Considering biphasic preparation methods, median sample preparation peak area RSDs (1 min partition time) were 8.9–9.2% and 7.9–8.2% for positive and negative ion, respectively (Fig. 3) and median UHPLC-MS injection peak area RSDs were 6.2–8.7% and 6.5–7.1% for positive and negative ion, respectively (Fig. S11†). Along with the PCA scores plot (Fig. S12†), this demonstrates low sample preparation and injection technical variance similar to the monophasic methods. The biphasic method (1 min partition time) with the most detected putative metabolites was MTBE : MeOH : H_2_O in positive (1136) and negative ion modes (736), followed by DCM : MeOH : H_2_O (924 pos., 705 neg.) and CHCl_3_ : MeOH : H_2_O (852 pos., 707 neg.). With the exception of DCM : MeOH : H_2_O and CHCl_3_ : MeOH : H_2_O in positive ion mode, these values were similar to the monophasic methods. The lower detection response of DCM : MeOH : H_2_O and CHCl_3_ : MeOH : H_2_O in positive ion mode was attributed to decreased lipid detection response (Fig. 4; visible in the UHPLC-MS chromatogram, ∼RT 4.2 min, Fig. S13A†), indicating these methods are the best for lipid removal from the polar phase. This is noteworthy because phospholipid removal from polar extracts can lower ionisation suppression and ion competition during UHPLC-MS analysis.^24^ The high polar metabolite yield of MTBE : MeOH : H_2_O relative to the other biphasic methods is consistent with previous observations,^13^ and may be due to the lower polarity of MTBE relative to chloroform and dichloromethane causing a greater number or concentration of polar compounds (and polar lipids, Fig. 4) to partition into the aqueous phase. Considering polar metabolites, there was a similar detection response across the three biphasic methods (Fig. 2 and Table S1†). Increasing CHCl_3_ : MeOH : H_2_O partition time increased the polar metabolite and very polar lipid (*e.g.* acyl carnitines) responses in the polar extract (Table S2†). Increasing partition time for MTBE : MeOH : H_2_O and DCM : MeOH : H_2_O methods, decreased the lipid detection response in the polar extract (Table S2†). These small changes indicate that the longer partition step allows phase-partition to get closer to equilibrium.

We conclude that monophasic 50 : 50 ACN : MeOH is the optimal method in terms of reproducibility and number of detected putative metabolites. As a monophasic method it is a shorter, quicker and simpler procedure than biphasic methods. Plasma–solvent incubation is not recommended as (i) there is no reproducibility or yield improvement, and (ii) it can enhance lipid extraction, which is not necessarily desirable in polar sample preparations. CHCl_3_ : MeOH : H_2_O is the recommended biphasic method as it provides the best lipid removal from the polar extract, while also providing good reproducibility and detection sensitivity. A 20 min partition is recommended as it allows phase-partitioning to reach equilibrium.

### 3.4. Optimised plasma sample preparation for the C_18_ reversed phase lipids UHPLC-MS assay

Monophasic sample preparation methods containing some isopropanol (25 : 75 ACN : IPA, 50 : 50 ACN : IPA, 75 : 25 ACN : IPA, 100% IPA) were highly reproducible (Fig. 3). For these methods (with no solvent incubation) median sample preparation peak area RSDs were 4.7–6.9% and 4.5–6.6% for positive and negative ion, respectively (Fig. 3), and median UHPLC-MS injection peak area RSDs were 3.9–4.3% and 4.1–4.7% for positive and negative ion, respectively (Fig. S11†), demonstrating minimal technical variance arising from sample preparation and sample analysis. PCA scores plots (Fig. S14†) provide further evidence of high reproducibility and showed that the higher the IPA content, the higher the method reproducibility (Fig. S14†). 100% ACN had higher median sample preparation peak area RSDs (no solvent incubation: positive ion 10.3%; negative ion 12.4%) than IPA-based methods, indicating greater sample preparation technical variance. Lipid classes that had poor reproducibility in the 100% ACN included ceramides, some phosphatidylcholines and some triglycerides (Table S1†). Ceramides and triglycerides are very non-polar lipids, suggesting 100% ACN is not suitable for the extraction of very non-polar compounds.

**Fig. 5:**
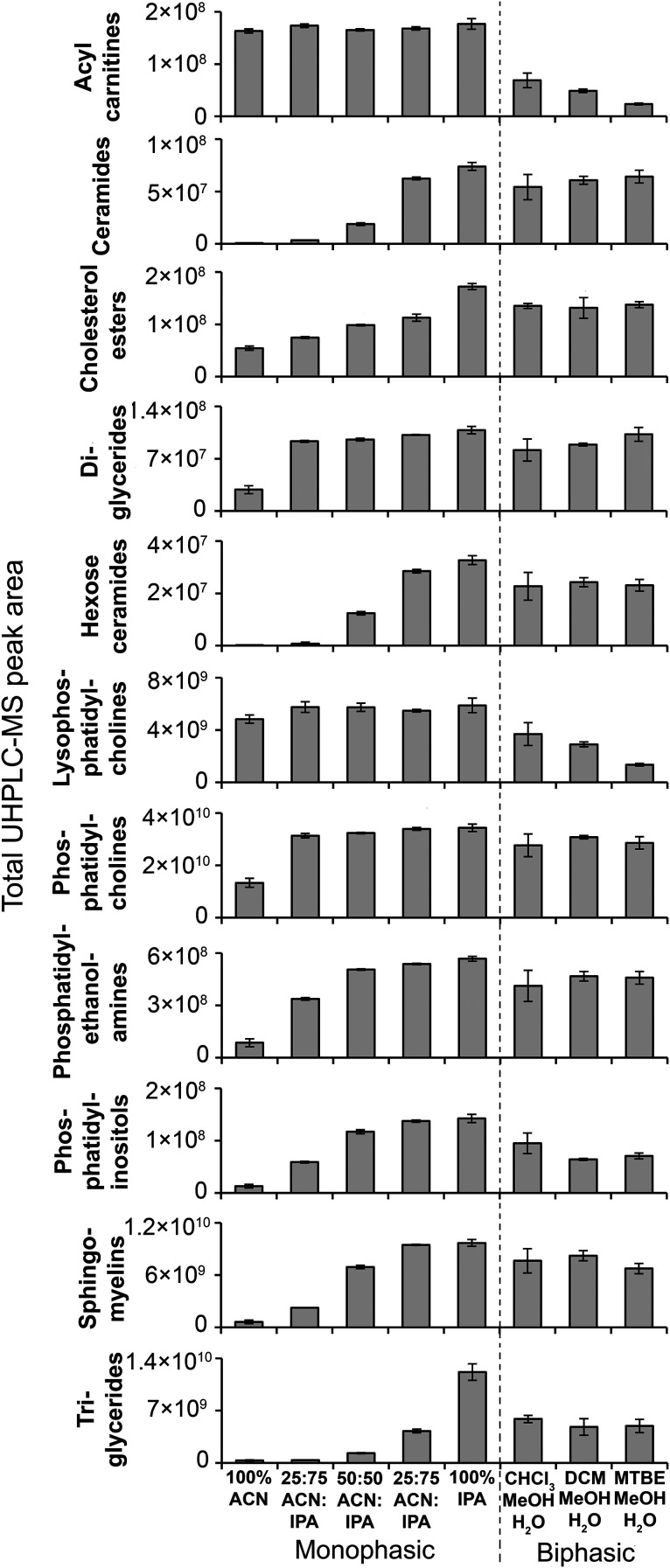
MS/MS-annotated lipids detected in the C_18_ UHPLC-MS analysis of lipid extractions of plasma. UHPLC-MS positive ion total peak area for each annotated lipid class is shown as mean ± sd (*n* = 3 preparation replicates; no solvent incubation for monophasic and 1 min partition time for biphasic methods). Lipids were annotated from QC UHPLC-MS/MS data using Lipid Search software (*m*/*z*, retention times, MS/MS annotation grade, mean peak area and standard deviations for each lipid species is shown in Table S1†).

Considering putative metabolite and lipid detection, 25 : 75 ACN : IPA and 100% IPA had the highest for both positive (1680–1682 putative metabolites/lipids) and negative (839–845) ion modes (Fig. 3). The lipid detection response and number of detected putative metabolites/lipids increased with increasing IPA content (Fig. 3, Fig. 5 and Table S1;† also visible in the base peak chromatograms, Fig. S15†), consistent with previous findings.^12^ Incubation with extraction solvent had little effect on reproducibility or numbers of detected putative metabolites or lipids, except for 100% ACN where solvent incubation (60 min at 4 °C or 60 min at −20 °C) showed a small increase in reproducibility and numbers of detected putative metabolites/lipids (Fig. 3).

Biphasic methods (1 min partition time) had higher sample preparation variance than monophasic methods containing IPA: median sample preparation peak area RSDs were 8.5–21.5% and 7.4–24.6% (positive and negative ion, respectively; Fig. 3); and median UHPLC-MS injection peak area RSDs were 4.0–4.1% and 3.9–4.3% (positive and negative ion, respectively; Fig. S11†). The increased variance was likely caused by additional steps in biphasic extraction procedures (pipetting, drying and reconstitution) compared to monophasic procedures. Biphasic methods had a lower lipid detection response and detected fewer putatively annotated metabolites or lipids than monophasic methods (Fig. 3, 5 and Table S1†), consistent with previous observations.^12,16^ This is due to lower recovery of polar lipids which partition into both the polar and non-polar phases (Fig. 4 and 5) and potential loss of compounds during drying and reconstitution stages, steps that are not present in monophasic methods. Increasing CHCl_3_ : MeOH : H_2_O biphasic partition time (20 min) improved method reproducibility and detection of putatively annotated metabolites/lipids (as observed with tissues previously^14^), but increasing partition time had no improvement for the other biphasic methods (Fig. 3).

We conclude that monophasic 100% IPA is the preferred method because (i) it is the most reproducible method, (ii) it has the highest yield for all lipid classes, including both polar (*e.g.* acyl carnitines) and non-polar (*e.g.* triglycerides), and (iii) it is a simple and rapid method (Fig. 5). Plasma–solvent incubation does not improve yield or reproducibility so is not recommended. Where the sample volume is insufficient for separate polar and non-polar preparations, we recommend CHCl_3_ : MeOH : H_2_O biphasic (20 min partition) as it provides the highest lipid response for a biphasic method with an acceptable level of reproducibility (Fig. 3 and 5).

Considering the results from sections 3.3 and 3.4, biphasic methods had higher final extraction plasma : solvent ratios compared to monophasic methods (monophasic 1 : 3, biphasic methods with CHCl_3_ or DCM 1 : 17.6, biphasic methods with MTBE 1 : 21.4). However, following drying and reconstitution of biphasic samples, all injected samples from all methods had an equivalent plasma dilution. The higher extraction solvent volumes would be expected to improve extraction efficiency (more solvent space for compounds to partition into) and reproducibility (easier to pipette large volumes reproducibly); however even with these proposed benefits, biphasic methods generally performed worse than monophasic methods in terms of yield and reproducibility.

## 4. Conclusions

We compared multiple monophasic (urine and plasma) and biphasic (plasma) sample preparation methods for UHPLC-MS metabolic and lipid phenotyping, assessing methods in terms of reproducibility and yield. Yield was assessed by the numbers of detected putatively annotated metabolites (based on accurate mass) and the responses of MS/MS-annotated metabolites and lipids. Biphasic extracts were dried and reconstituted before UHPLC-MS analysis whereas monophasic preparations were directly injected on to the UHPLC column. This was done for two reasons: (i) biphasic lipid extracts were not suitable for direct injection onto UHPLC columns and (ii) it allowed us to accurately match plasma dilutions during monophasic and biphasic preparations. The recommended sample preparation methods for urine are monophasic 50 : 50 MeOH : H_2_O for C_18_ aqueous reversed phase assay and monophasic 50 : 50 ACN : MeOH for the HILIC assay (in both cases 1 : 3 urine/solvent; no plasma–solvent incubation). For each UHPLC-MS assay, these methods detected the highest number of putative metabolites, demonstrated high sample preparation reproducibility and were appropriate solvents for direct injection onto the UHPLC column. The recommended method for plasma polar HILIC UHPLC-MS metabolomics is monophasic 50 : 50 ACN : MeOH (1 : 3 plasma/solvent; no plasma–solvent incubation) as this detected the highest number of putative metabolites with high reproducibility. The biphasic CHCl_3_ : MeOH : H_2_O method (20 min partition) is recommended for plasma HILIC UHPLC-MS analysis where lipid removal from the polar extract was required. 100% IPA (1 : 3 plasma/solvent; no plasma–solvent incubation) is recommended for plasma C_18_ lipidomics due to high reproducibility and the highest detection responses for all measured lipid classes. Biofluid–solvent incubation during monophasic preparation provides no improvement in yield or reproducibility and is not required. The monophasic methods we recommend are simple, quick and high-throughput procedures ideal for large metabolic phenotyping studies. A limitation of the study is that isotopically labelled internal standard compounds were not used. Isotopically-labelled internal standard(s) can be used to check any change in reproducibility within each analysed sample for the exact non-isotopically labelled version of the specific internal standard(s) used (given that factors affecting reproducibility [*e.g.* matrix effects] are specific to compound retention time and will thus will be different for different compounds). Here, we assessed the reproducibility of multiple chemically diverse endogenous compounds (putative and MS/MS annotated) in three different ways: (i) repeat injections of a homogenous quality control (QC) sample at regular intervals throughout the analytical run to monitor analytical detection performance, (ii) for each sample preparation method we measured sample preparation replicates to measure the combined sample preparation and UHPLC-MS injection variance, and (iii) for each sample preparation method we measured UHPLC-MS injection reproducibility to measure the variance directly attributable to UHPLC-MS performance. Isotopically labelled internal standards are an easier and quicker way to define instrument variance *versus* sample preparation variance compared to our approach used here (analysis of separate sample preparation and UHPLC-MS injection replicates), therefore we would advise the use of isotopically labelled internal standards for this purpose in future biological studies.

## Abbreviations

UHPLC-MS: Ultra-high-performance liquid chromatography-mass spectrometry
HILIC: Hydrophilic interaction chromatography
MTBE: Methyl *tert*-butyl ether
MeOH: Methanol
ACN: Acetonitrile
IPA: Isopropanol
CHCl_3_: Chloroform
DCM: Dichloromethane
PCA: Principal components analysis
QC: Quality control
RSD: Relative standard deviation
MS/MS: Tandem mass spectrometry

## Author contributions

Andrew Southam: Conceptualization, Methodology, Formal analysis, Investigation, Writing – Original Draft, Supervision, Project administration. Liam Haglington: Conceptualization, Methodology, Investigation, Writing – Review & Editing. Lukáš Najdekr: Methodology, Investigation, Writing – Review & Editing. Andris Jankevics: Formal analysis, Software, Writing – Review & Editing. Ralf Weber: Software, Data Curation, Supervision, Writing – Review & Editing. Warwick Dunn: Conceptualization, Methodology, Writing – Original Draft, Supervision, Project administration.

## Conflicts of interest

The authors declare that they have no known competing financial interests or personal relationships that could have appeared to influence the work reported in this paper.

## Supplementary Material

AN-145-D0AN01319F-s001

AN-145-D0AN01319F-s002

AN-145-D0AN01319F-s003
