# Supplementary material for: Assessment of human plasma and urine sample preparation for reproducible and high-throughput UHPLC-MS clinical metabolic phenotyping
Source: Analyst. 2020 Jul 29;145(20):6511–23. doi: 10.1039/d0an01319f (PMC13444864; doi:10.1039/d0an01319f)
Supplement: Supplementary file 1 [file AN-145-D0AN01319F-s001.pdf]

Electronic Supplementary Material (ESI) for Analyst.
This journal is © The Royal Society of Chemistry 2020

Assessment of human plasma and urine sample preparation for reproducible and high-throughput UHPLC-MS clinical metabolic phenotyping

Supporting Information: Supporting Figures

Andrew D. Southam[1,2*], Liam D. Haglington L [1], Lukáš Najdekr[1,2], Andris Jankevics[1,2], Ralf J. M. Weber[1,2], Warwick B. Dunn[1,2,3]

[1]School of Biosciences, University of Birmingham, Edgbaston, Birmingham, B15 2TT, UK

[2]Phenome Centre Birmingham, University of Birmingham, Edgbaston, Birmingham, B15 2TT, UK

[3]Institute of Metabolism and Systems Research, University of Birmingham, Edgbaston, Birmingham, B15 2TT, UK

*Corresponding author: a.d.southam@bham.ac.uk

Supporting Tables and Figures

Table S1 (see associated Excel file): Polar metabolites and lipids annotated from MS/MS data in all assays. Information shown includes compound retention time, *m/z*, mean peak area, peak area standard deviation, peak RSDs, MS/MS identification grades.

Table S2 (see associated Excel file): Variance induced by solvent-biofluid incubation for MS/MS annotated polar metabolites and lipids across all assays.

		UHPLC-MS assay			
		C_{18} (aq) reversed phase – positive ion	C_{18} (aq) reversed phase – negative ion	HILIC – positive ion	HILIC – negative ion
Urine sample preparation solvent	100% ACN	0 (0%)	0 (0%)	7 (0.21%)	0 (0%)
	50:50 ACN:H_2O	0 (0%)	0 (0%)	0 (0%)	0 (0%)
	100% MeOH	1 (0.03%)	2 (0.04%)	0 (0%)	20 (0.59%)
	50:50 MeOH:H_2O	1 (0.03%)	2 (0.04%)	3 (0.09%)	2 (0.06%)
	50:50 ACN:MeOH	18 (0.5%)	4 (0.08%)	2 (0.06%)	0 (0%)
Total number of peaks present in the filtered data matrix		3482	4730	3301	3377

Table S3: The stability of urine samples post-preparation was high for all methods. For each sample preparation method the 3 extraction conditions (no incubation, 60 min incubation at 4°C, 60 min incubation at -20°C) were compared statistically (un-normalised data, tested by one-way ANOVA and corrected for multiple hypotheses tests, FDR<5%; done by MetaboAnalyst 4.0, https://www.metaboanalyst.ca/). In the table above the number of features (and % of total features in the dataset) from each of the final filtered datasets that changes significantly as a result of incubation are shown.

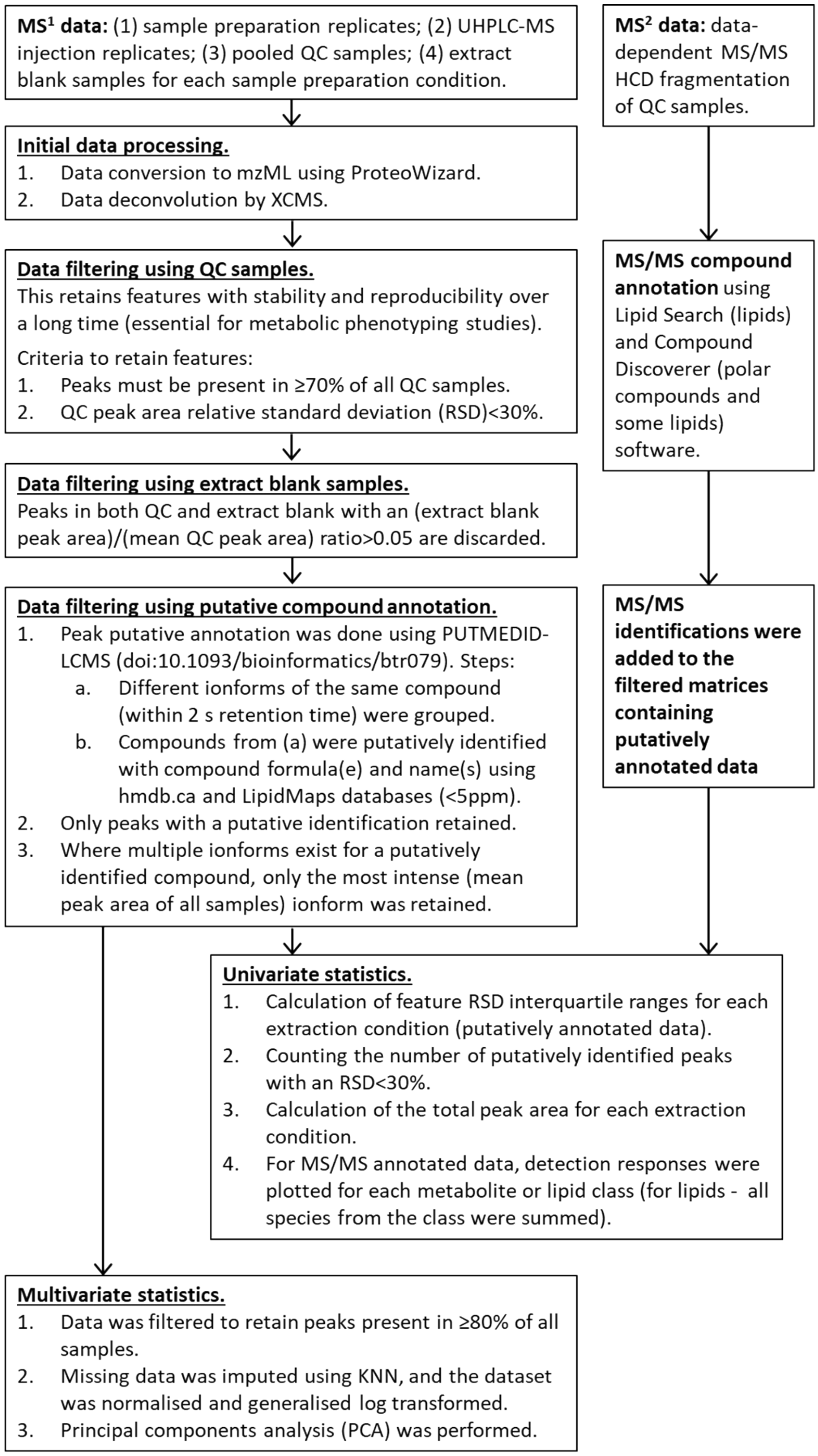

Figure S1: The processing pipeline for all data in this study.

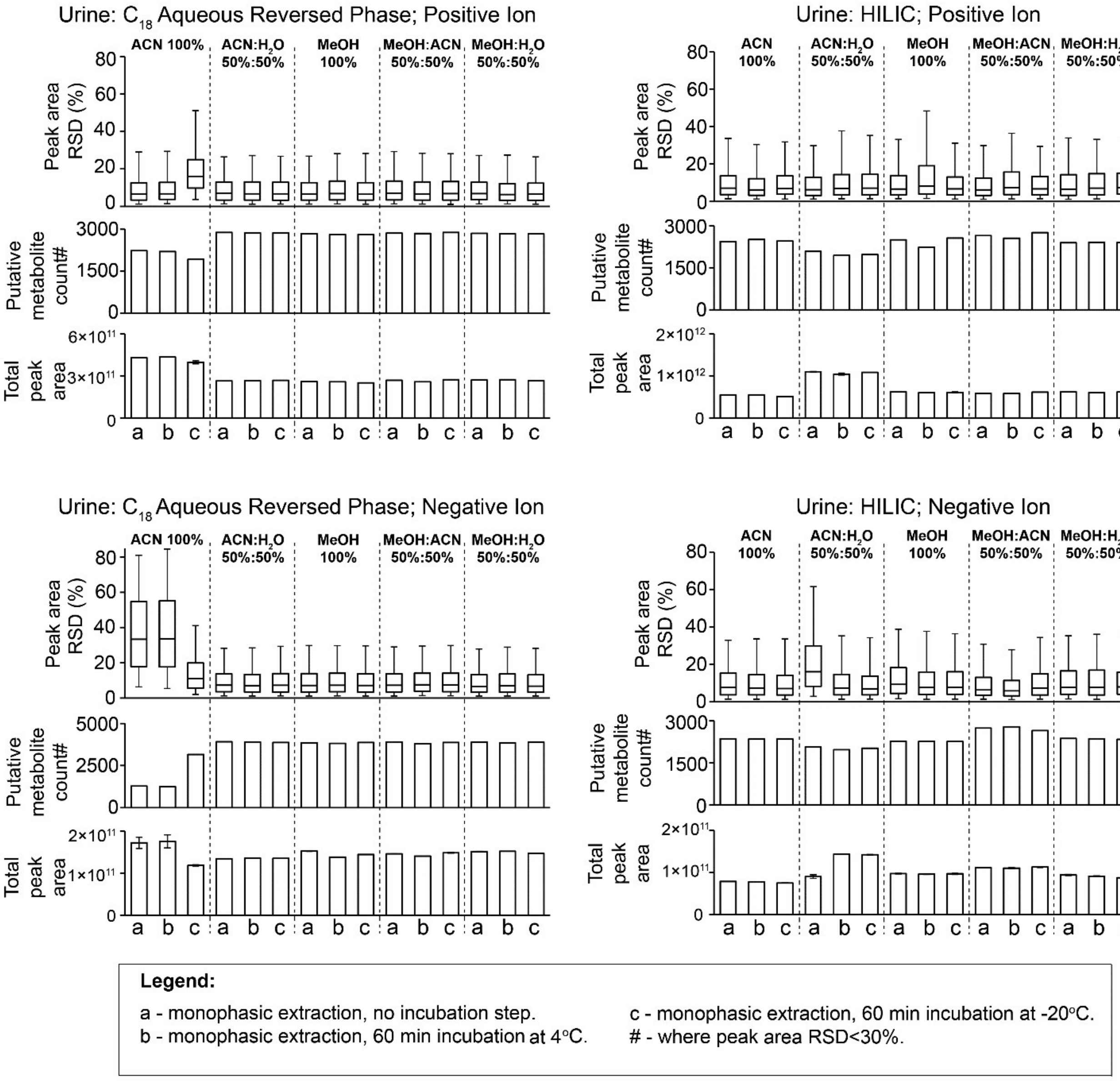

Figure S2: UHPLC-MS injection replicate data for putatively annotated metabolites from all urine sample preparation methods. Data was filtered using QC samples and then putatively annotated (Experimental Section). Relative standard deviations (RSD; shown as interquartile ranges and error bars as the 95th percentile) are calculated on peak intensities where the putative metabolite is present in all three sample preparation replicates. Putatively annotated metabolite counts are those present in three sample preparation replicates with an intensity RSD<30%.

Urine C_{18} (aq) reversed phase UHPLC-MS Data

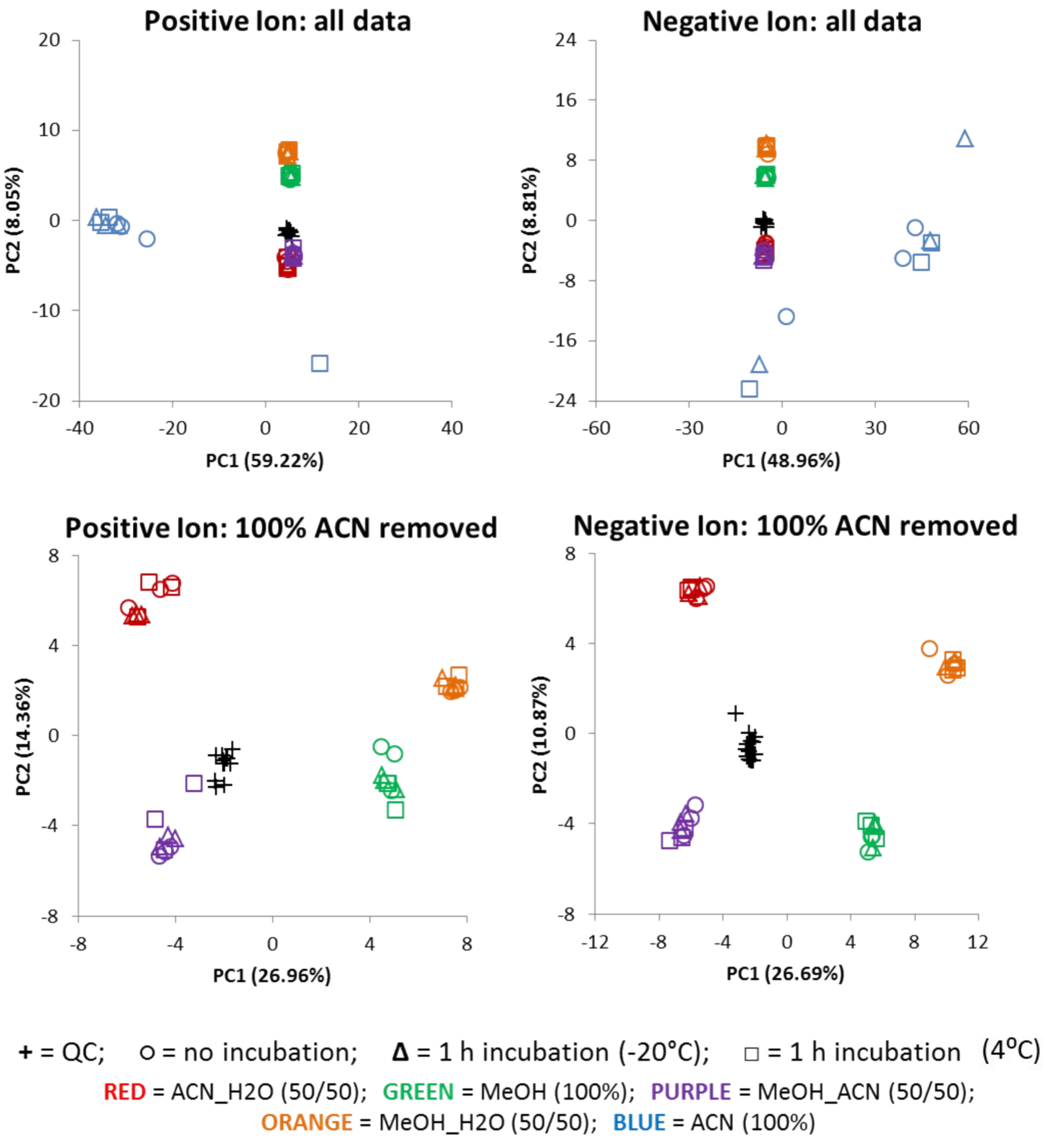

Figure S3: Principal components analysis (PCA) scores plots of the sample preparation replicates of each urine extraction method analysed by C_{18} aqueous reversed phase UHPLC-MS analysis.

Negative ion C_{18} aqueous reversed phase analysis of urine samples prepared by 100% ACN monophasic method with no solvent-biofluid incubation

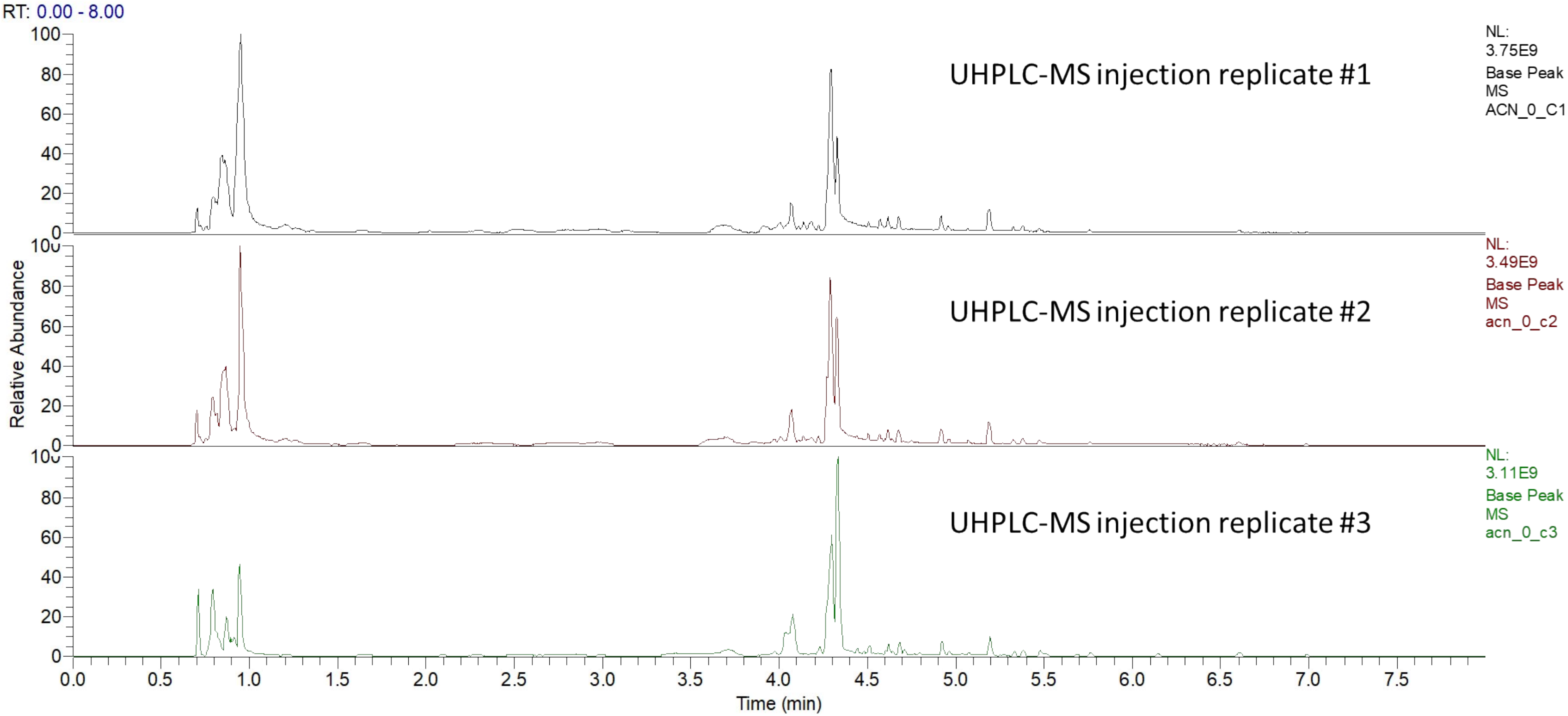

Figure S4 (A): Negative ion C_{18} aqueous reversed phase UHPLC-MS base peak chromatograms (mass range 100-1500 m/z) for urine samples prepared by 100% ACN monophasic method (with no solvent incubation).

Negative ion C_{18} aqueous reversed phase analysis of urine samples prepared by 100% ACN monophasic method with 60 min solvent-biofluid incubation (4^oC)

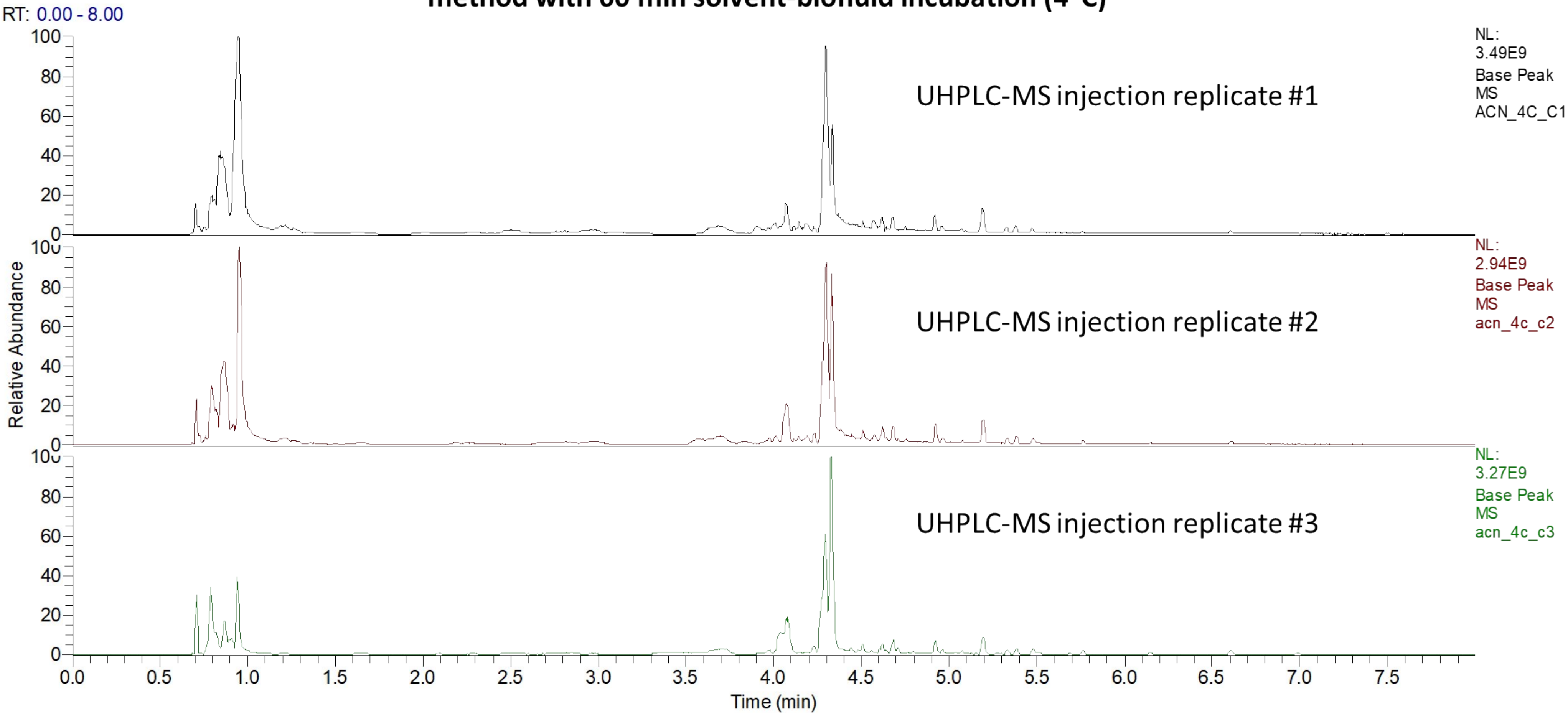

Figure S4 (B): Negative ion C_{18} aqueous reversed phase UHPLC-MS base peak chromatograms (mass range 100-1500 m/z) for urine samples prepared by 100% ACN monophasic method (60 min solvent incubation at 4^oC).

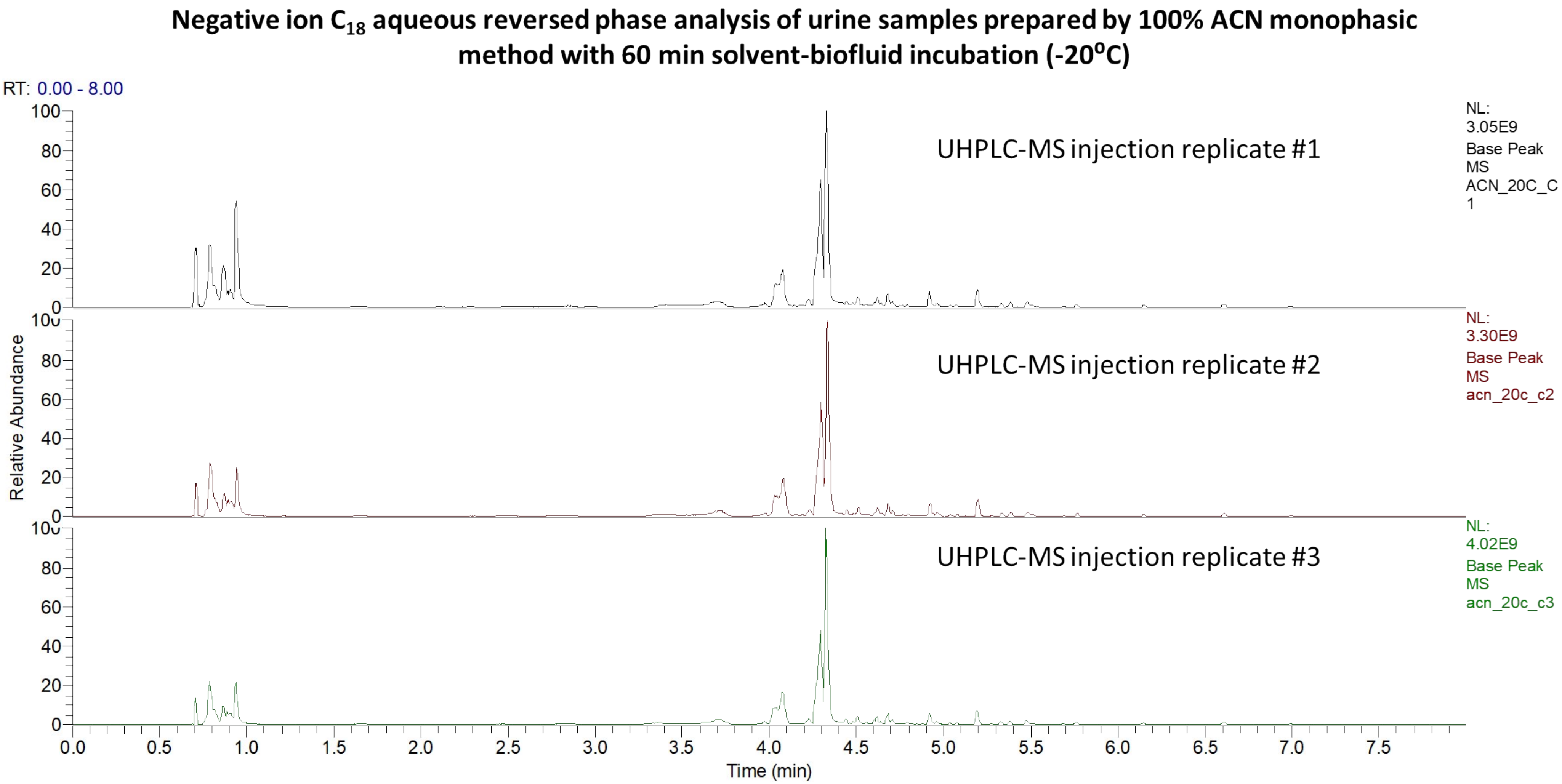

Figure S4 (C): Negative ion C_{18} aqueous reversed phase UHPLC-MS base peak chromatograms (mass range 100-1500 m/z) for urine samples prepared by 100% ACN monophasic method (60 min solvent incubation at -20°C).

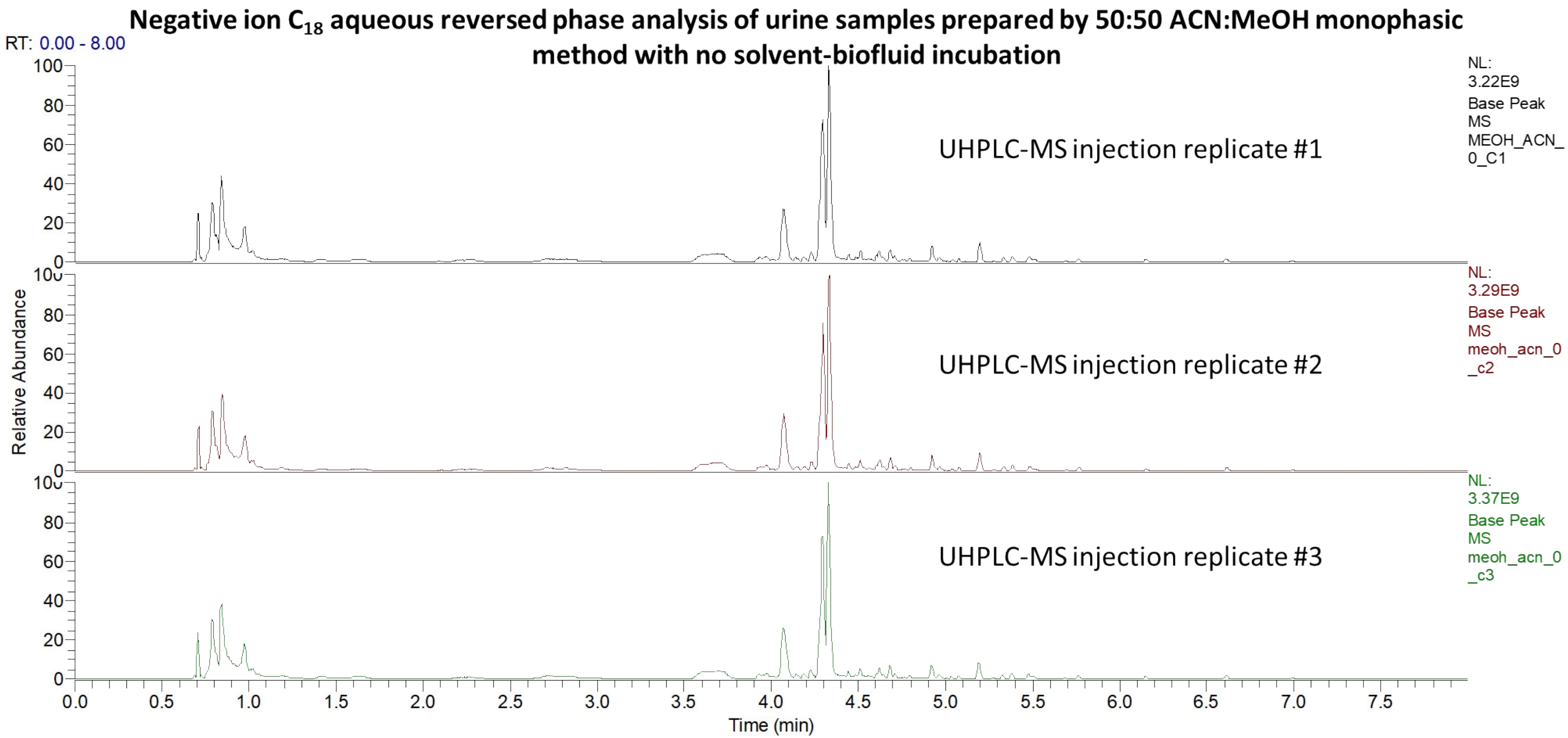

Figure S4 (D): Negative ion C_{18} aqueous reversed phase UHPLC-MS base peak chromatograms (mass range 100-1500 m/z) for urine samples prepared by 50:50 ACN:MeOH monophasic method (no solvent incubation).

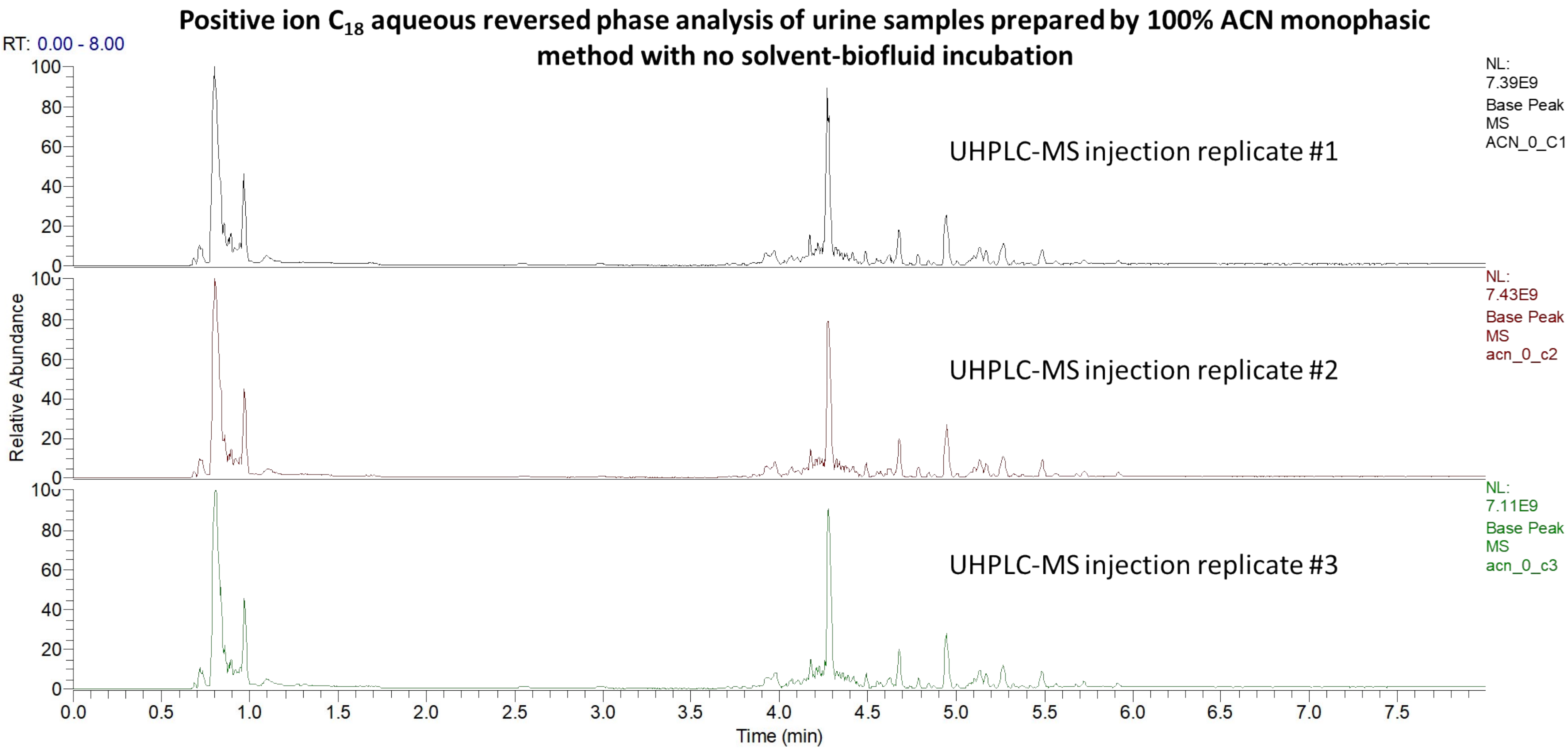

Figure S4 (E): Positive ion C_{18} aqueous reversed phase UHPLC-MS base peak chromatograms (mass range 100-1500 m/z) for urine samples prepared by 100% ACN monophasic method (no solvent incubation).

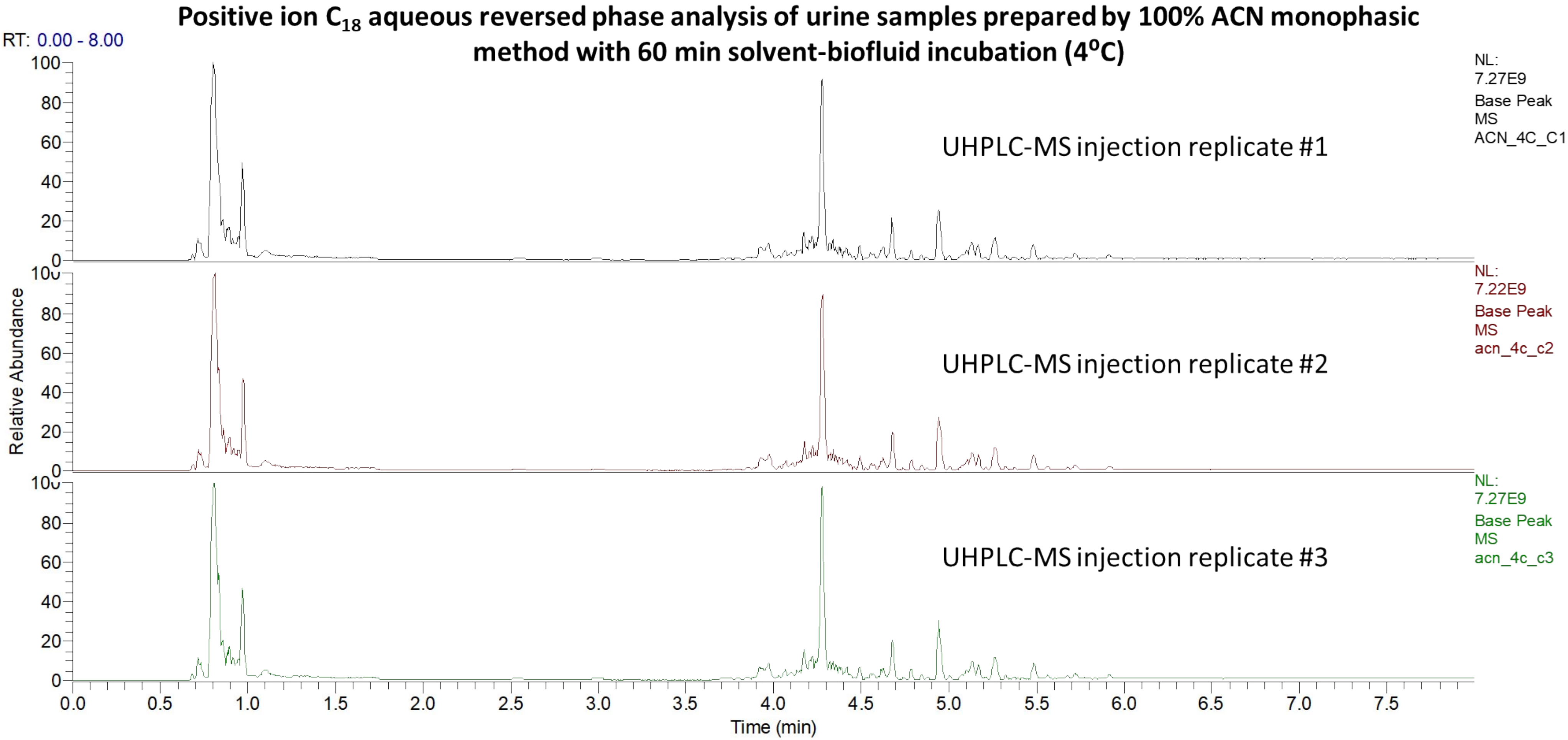

Figure S4 (F): Positive ion C_{18} aqueous reversed phase UHPLC-MS base peak chromatograms (mass range 100-1500 m/z) for urine samples prepared by 100% ACN monophasic method (60 min solvent incubation at 4^oC).

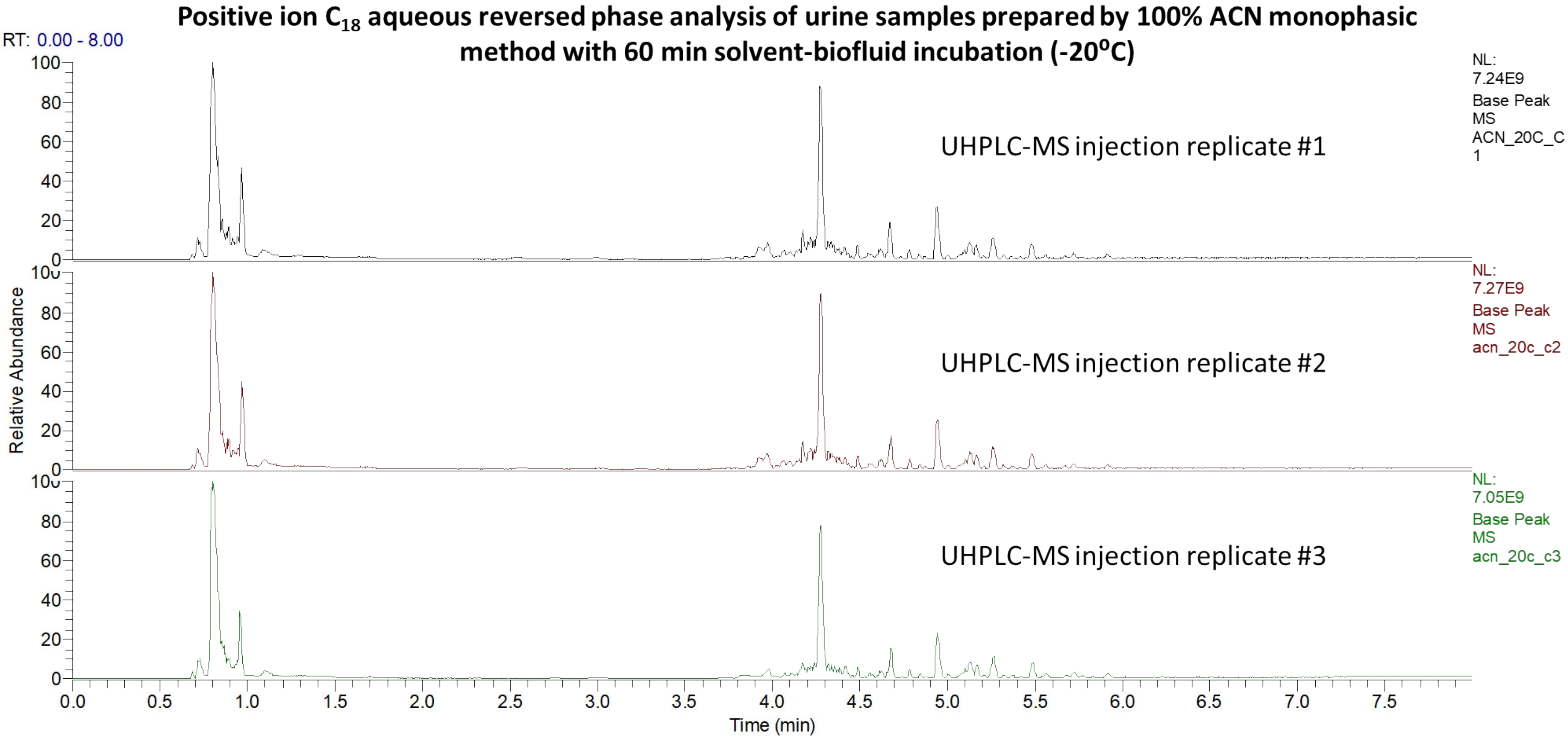

Figure S4 (G): Positive ion C_{18} aqueous reversed phase UHPLC-MS base peak chromatograms (mass range 100-1500 m/z) for urine samples prepared by 100% ACN monophasic method (60 min solvent incubation at -20°C).

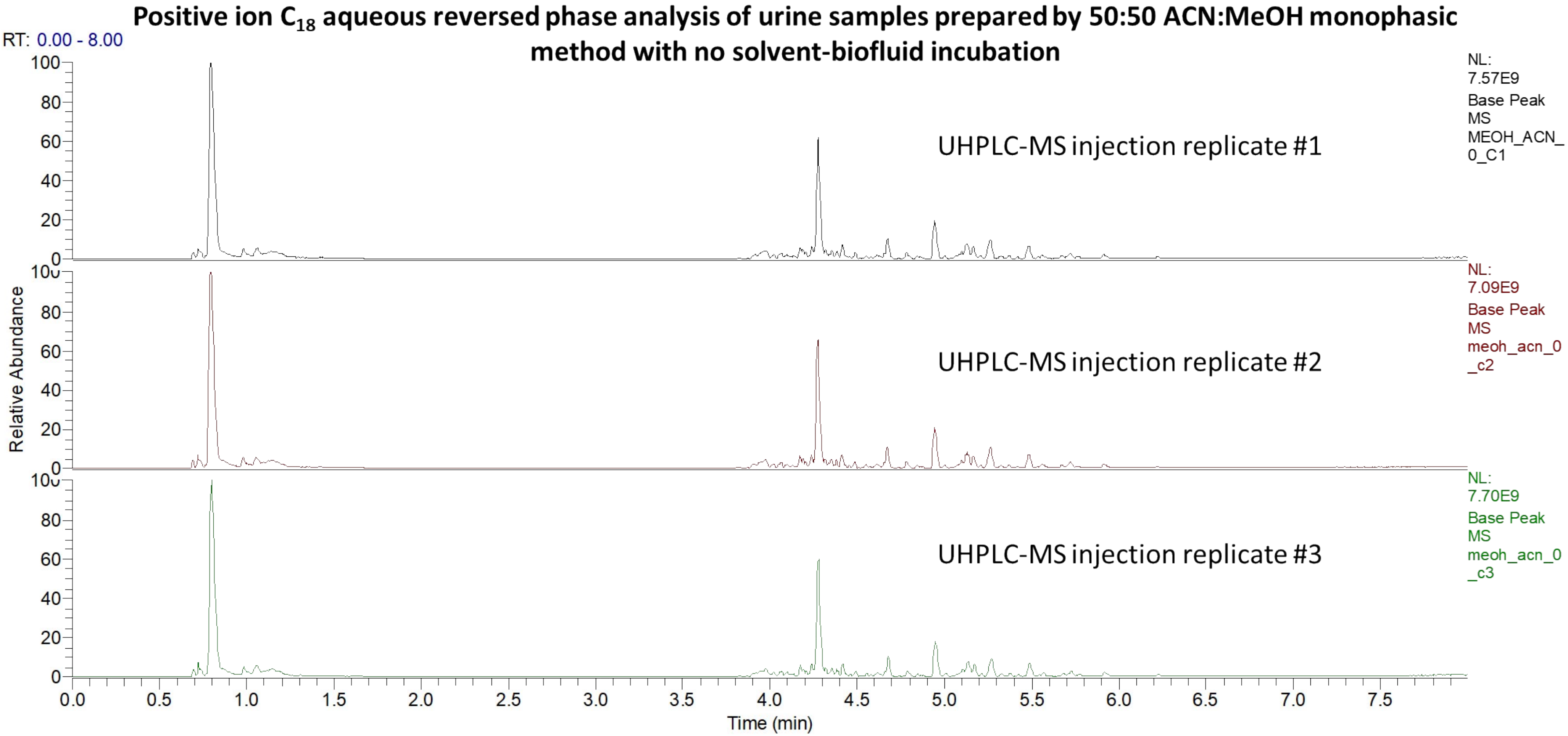

Figure S4 (H): Positive ion C_{18} aqueous reversed phase UHPLC-MS base peak chromatograms (mass range 100-1500 m/z) for urine samples prepared by 50:50 ACN:MeOH monophasic method (no solvent incubation).

Negative ion C_{18} aqueous reversed phase analysis of urine samples: base peak chromatograms for 191.0197 m/z (putatively annotated as $[citrate-H]^-$ or $[isocitrate-H]^-$)

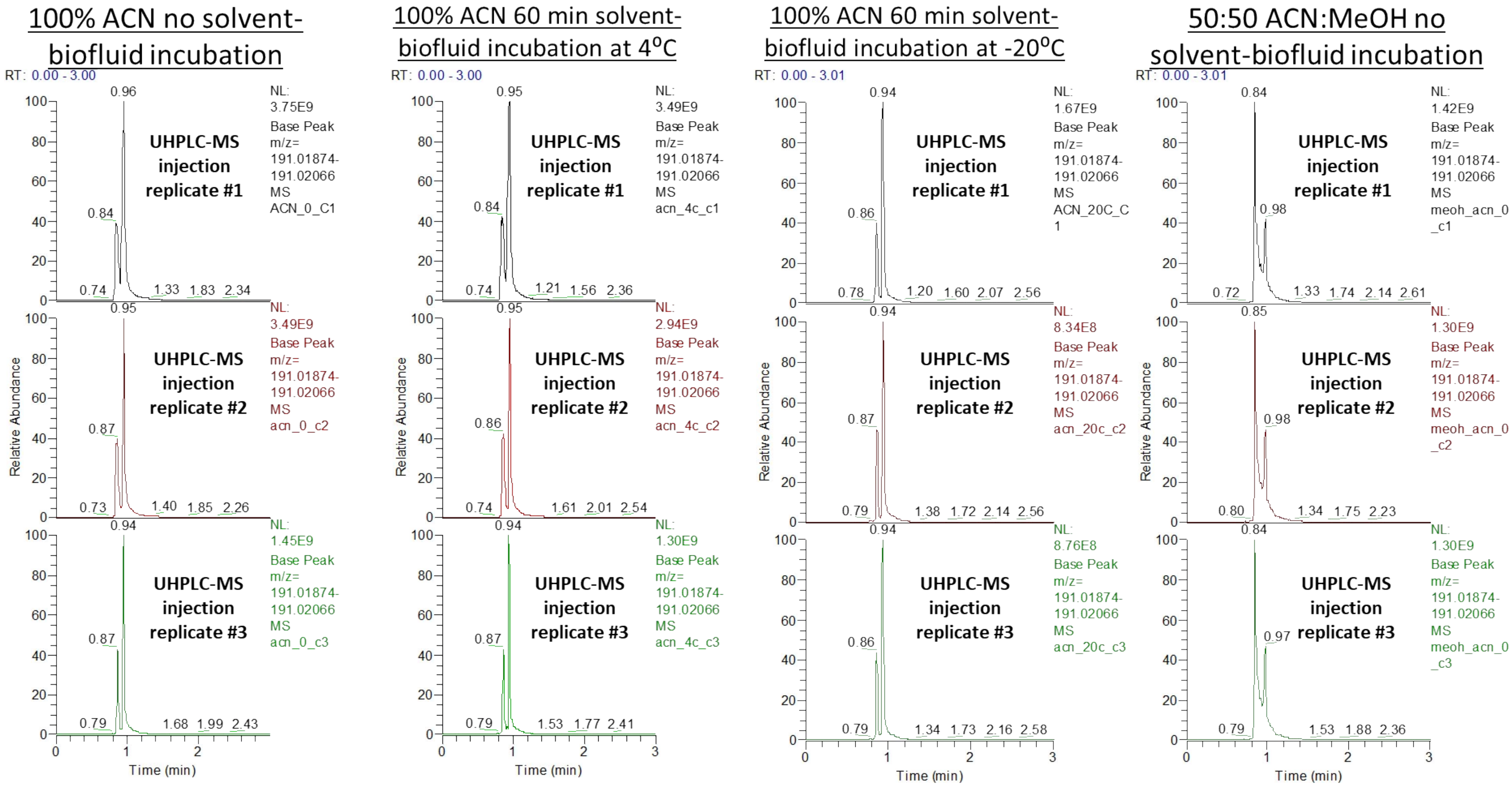

Figure S4 (I): Negative ion C_{18} aqueous reversed phase UHPLC-MS base peak chromatograms for the peak at 191.0197 m/z (putatively identified as citrate or isocitrate) for urine samples prepared by the indicated methods.

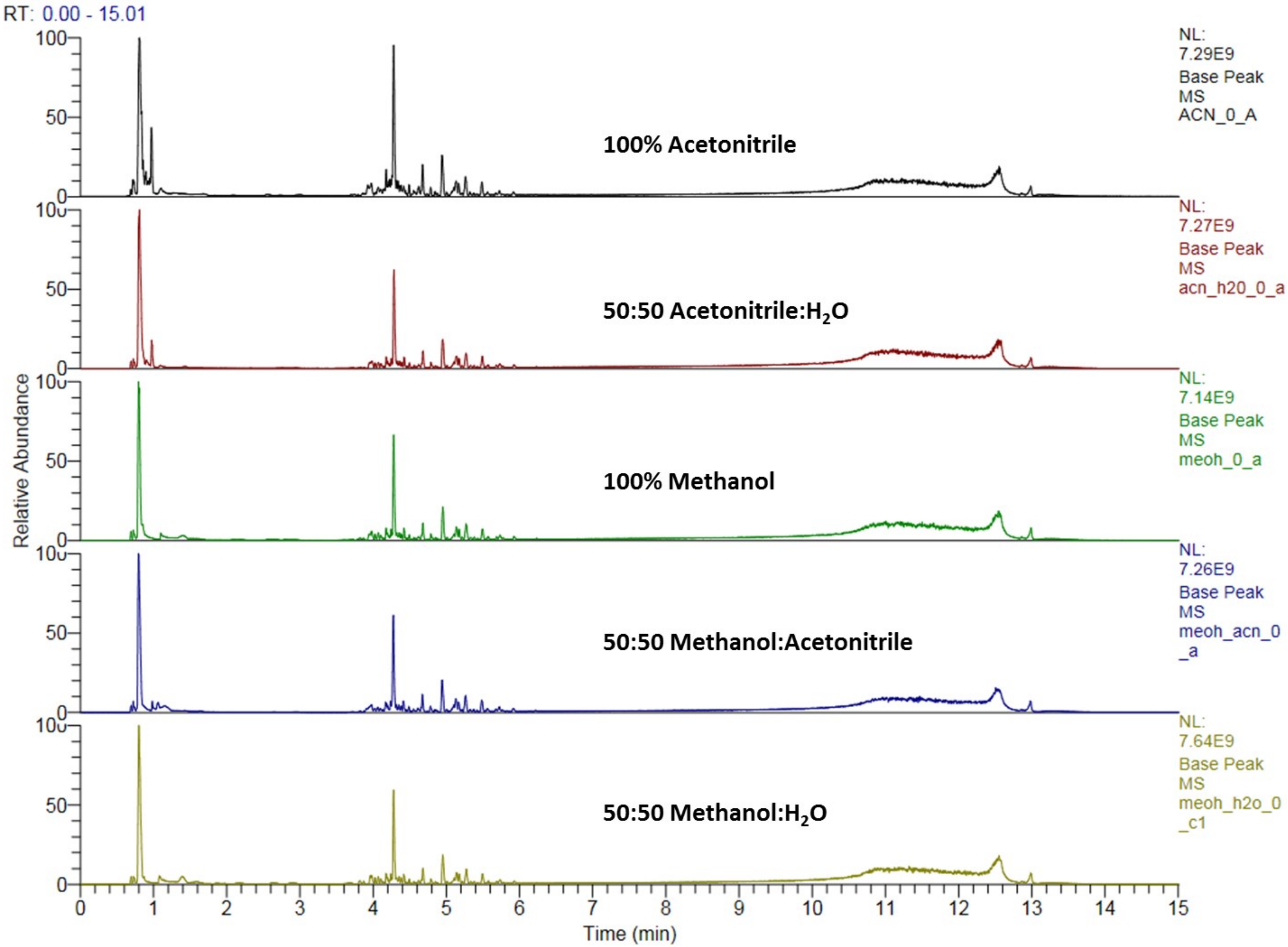

Figure S5 (A): Positive ion C_{18} aqueous reversed phase UHPLC-MS base peak chromatograms (mass range 100-1500 m/z) for the five monophasic urine extractions (with no solvent incubation).

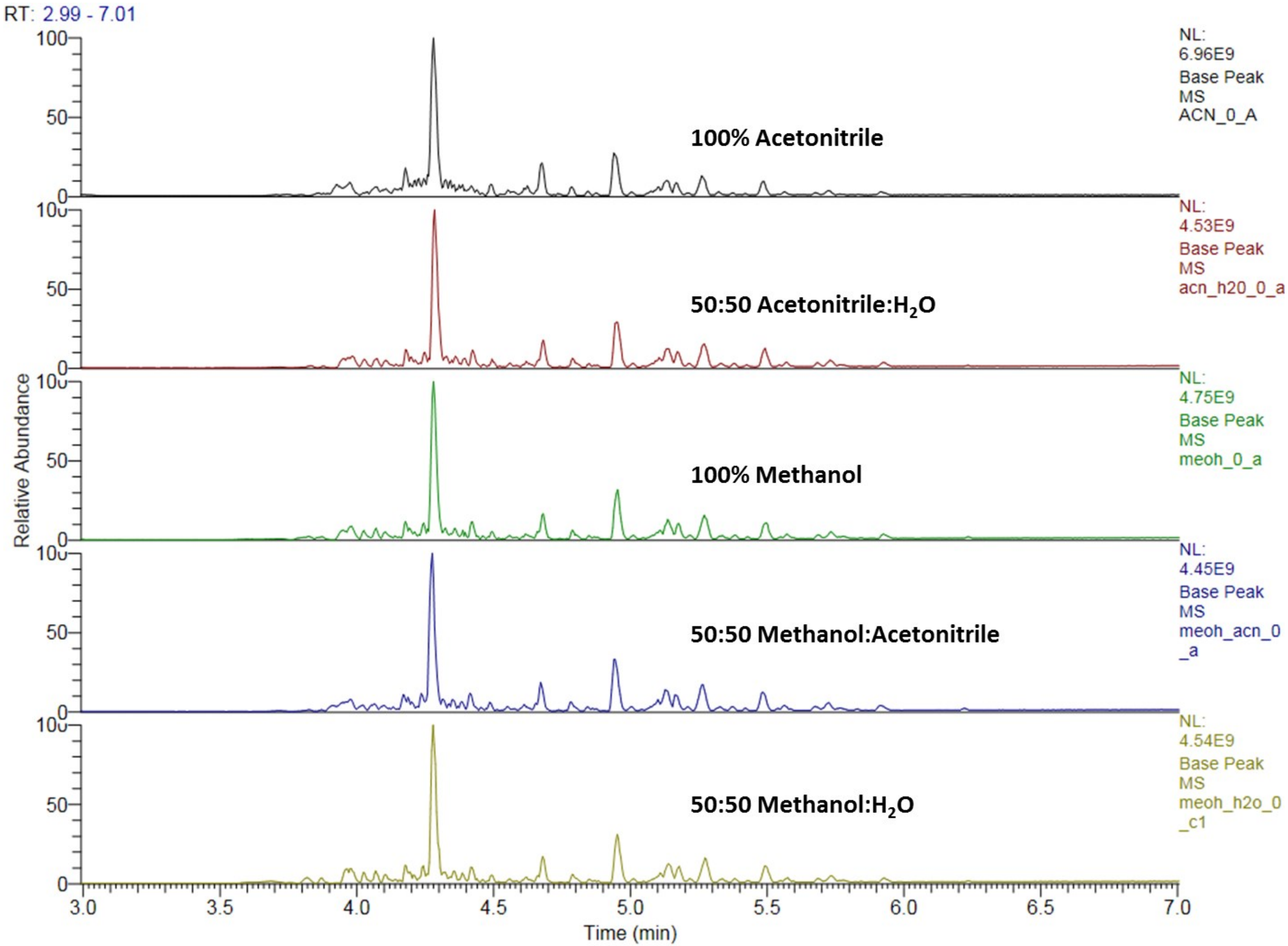

Figure S5 (B): Positive ion C_{18} aqueous reversed phase UHPLC-MS base peak chromatograms (mass range 100-1500 m/z) for the five monophasic urine extractions (with no solvent incubation). Zoomed on the retention time axis to show RT 3-7 min.

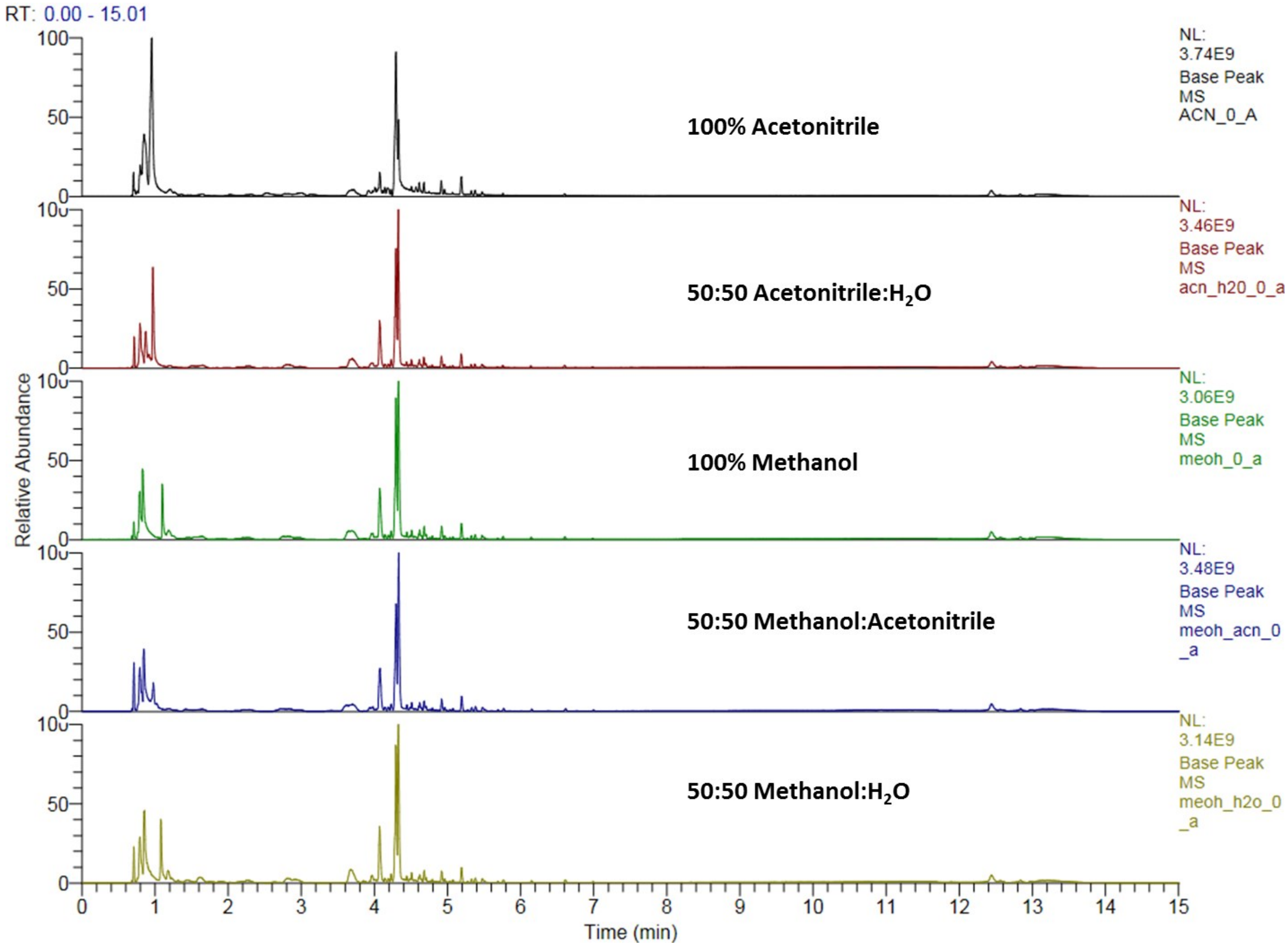

Figure S5 (C): Negative ion C_{18} aqueous reversed phase UHPLC-MS base peak chromatograms (mass range 100-1500 m/z) for the five monophasic urine extractions (with no solvent incubation).

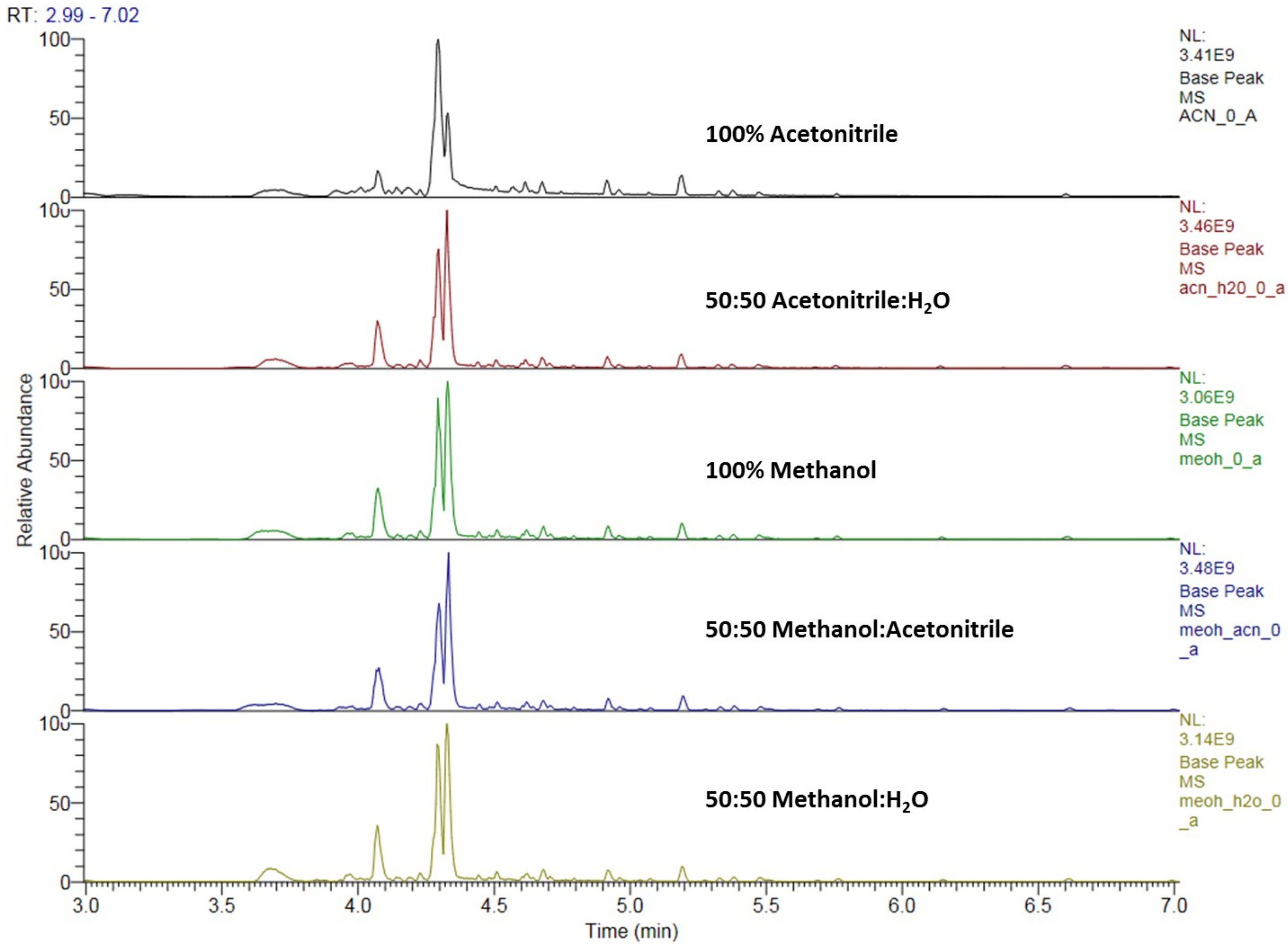

Figure S5 (D): Negative ion C_{18} aqueous reversed phase UHPLC-MS base peak chromatograms (mass range 100-1500 m/z) for the five monophasic urine extractions (with no solvent incubation). Zoomed on the retention time axis to show RT 3-7 min.

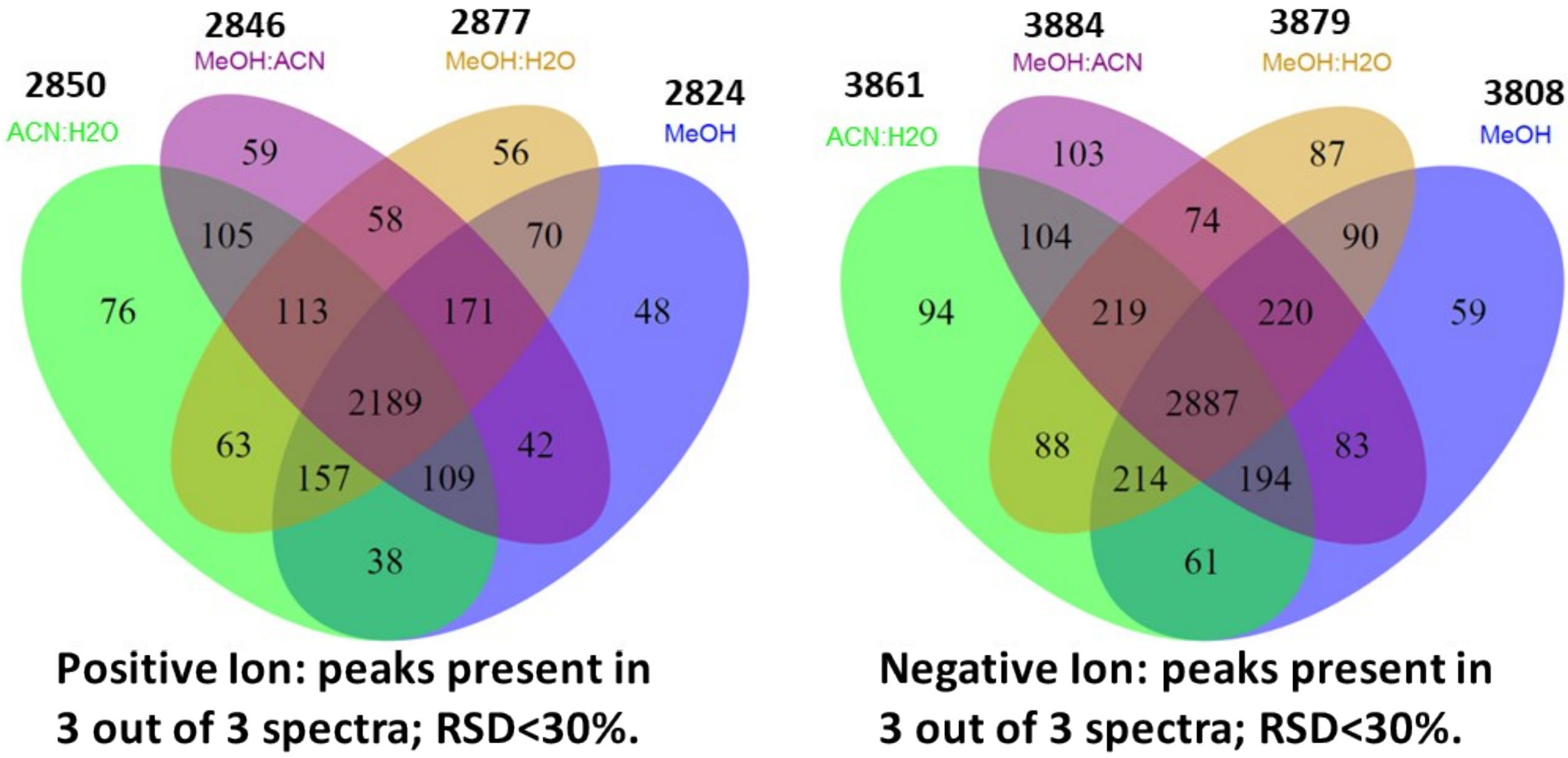

Figure S6: Venn diagrams showing the overlap in detected features across the four reproducible monophasic urine extraction methods analysed by C_{18} aqueous reversed phase UHPLC-MS (extraction replicate data, no solvent incubations). The number above each method indicates the total number of features detected by that method. The Venn diagram was conducted on data where peaks were present in 3 out of 3 extraction triplicates where peak intensity RSD<30%. Key: ACN = acetonitrile; MeOH = methanol

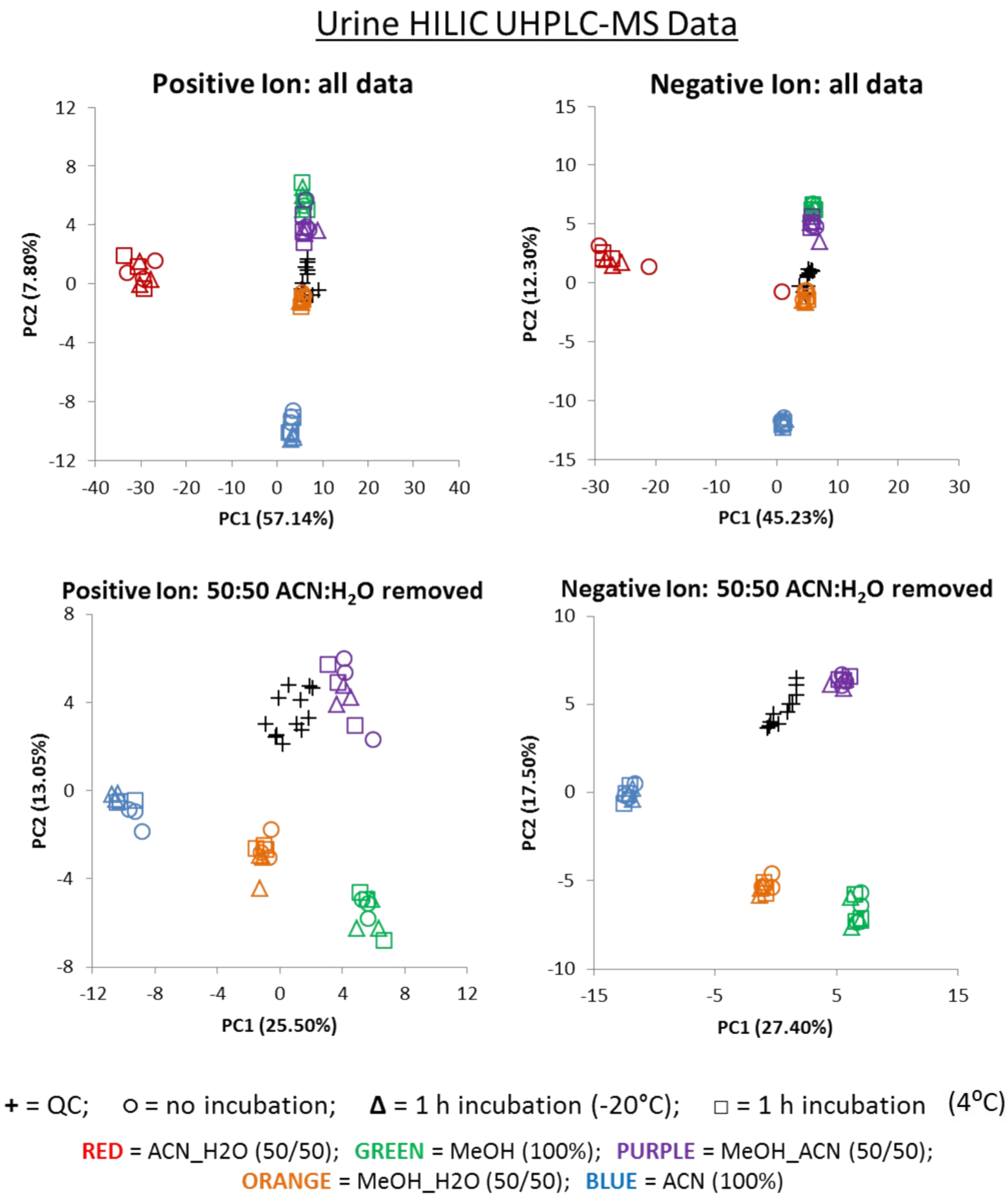

Figure S7: Principal components analysis (PCA) scores plots of the sample preparation replicates of each urine extraction method analysed by HILIC UHPLC-MS analysis.

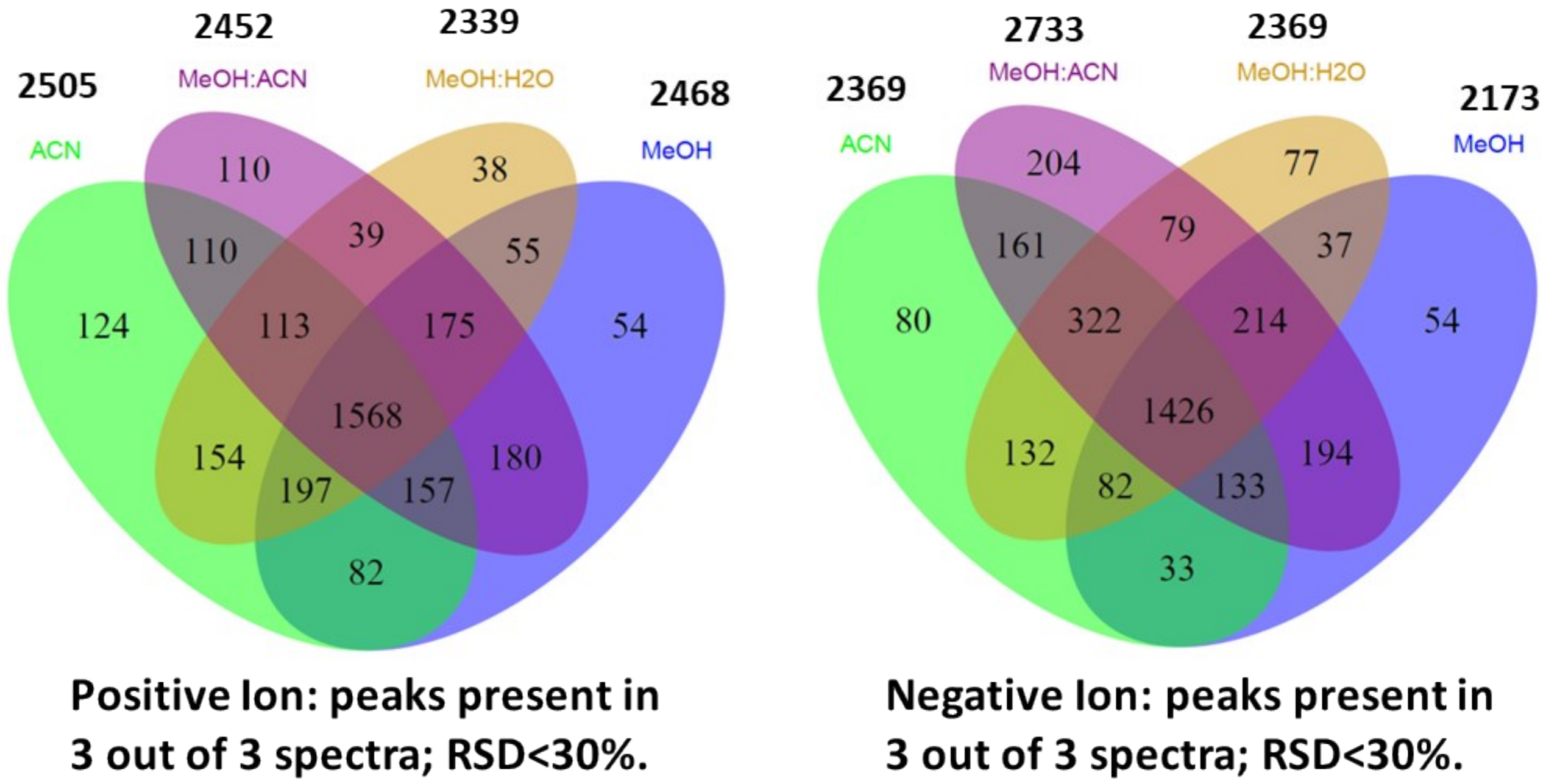

Figure S8: Venn diagrams showing the overlap in detected features across the four reproducible monophasic urine extraction methods analysed by HILIC UHPLC-MS (extraction replicate data, no solvent incubations). The number above each method indicates the total number of features detected by that method. The Venn diagram was conducted on where peaks were present in 3 out of 3 extraction triplicates where peak intensity RSD<30%. Key: ACN = acetonitrile; MeOH = methanol.

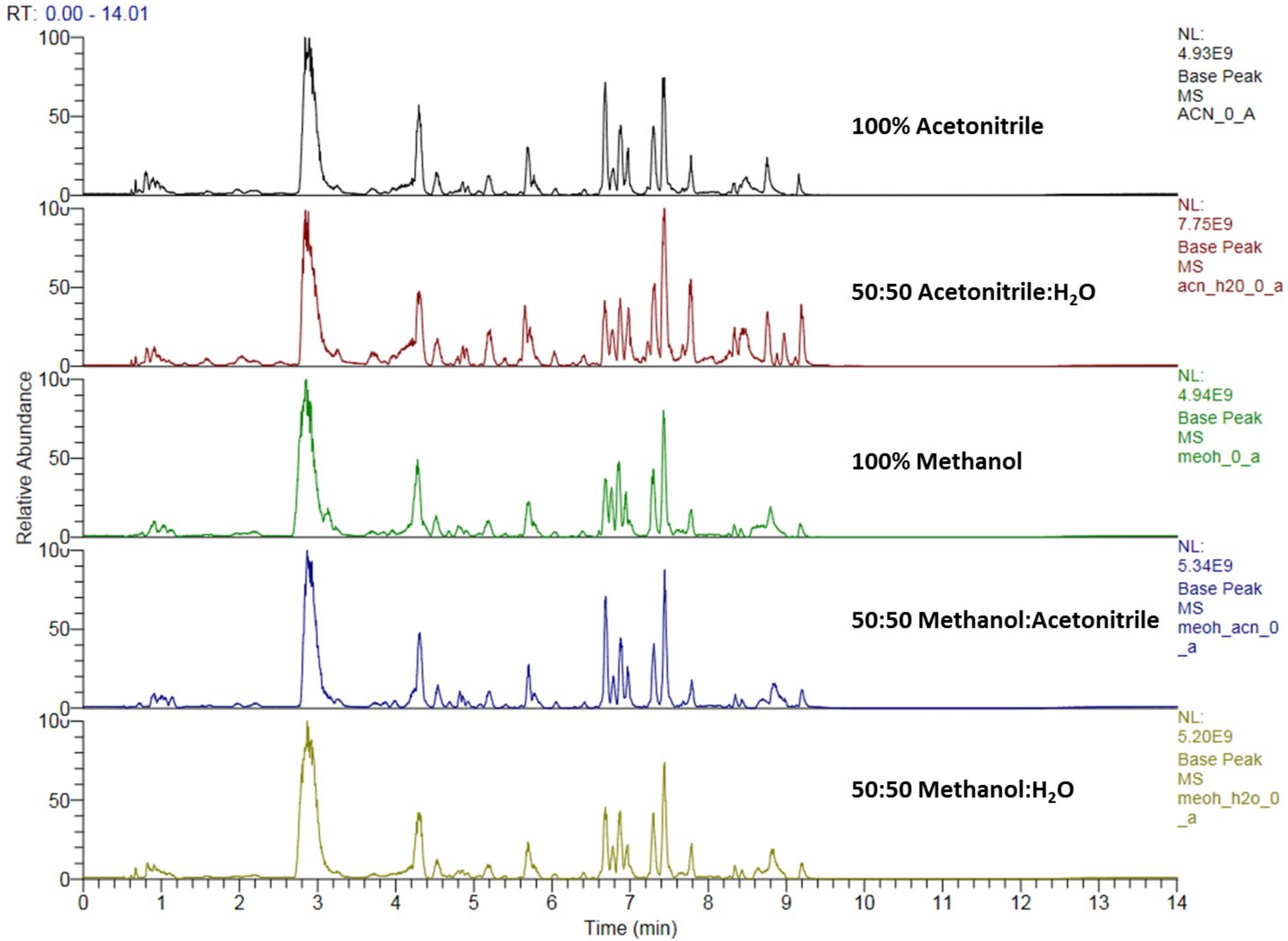

Figure S9 (A): Positive ion HILIC UHPLC-MS base peak chromatograms (mass range 70-1050 m/z) for the five monophasic urine extractions (with no solvent incubation).

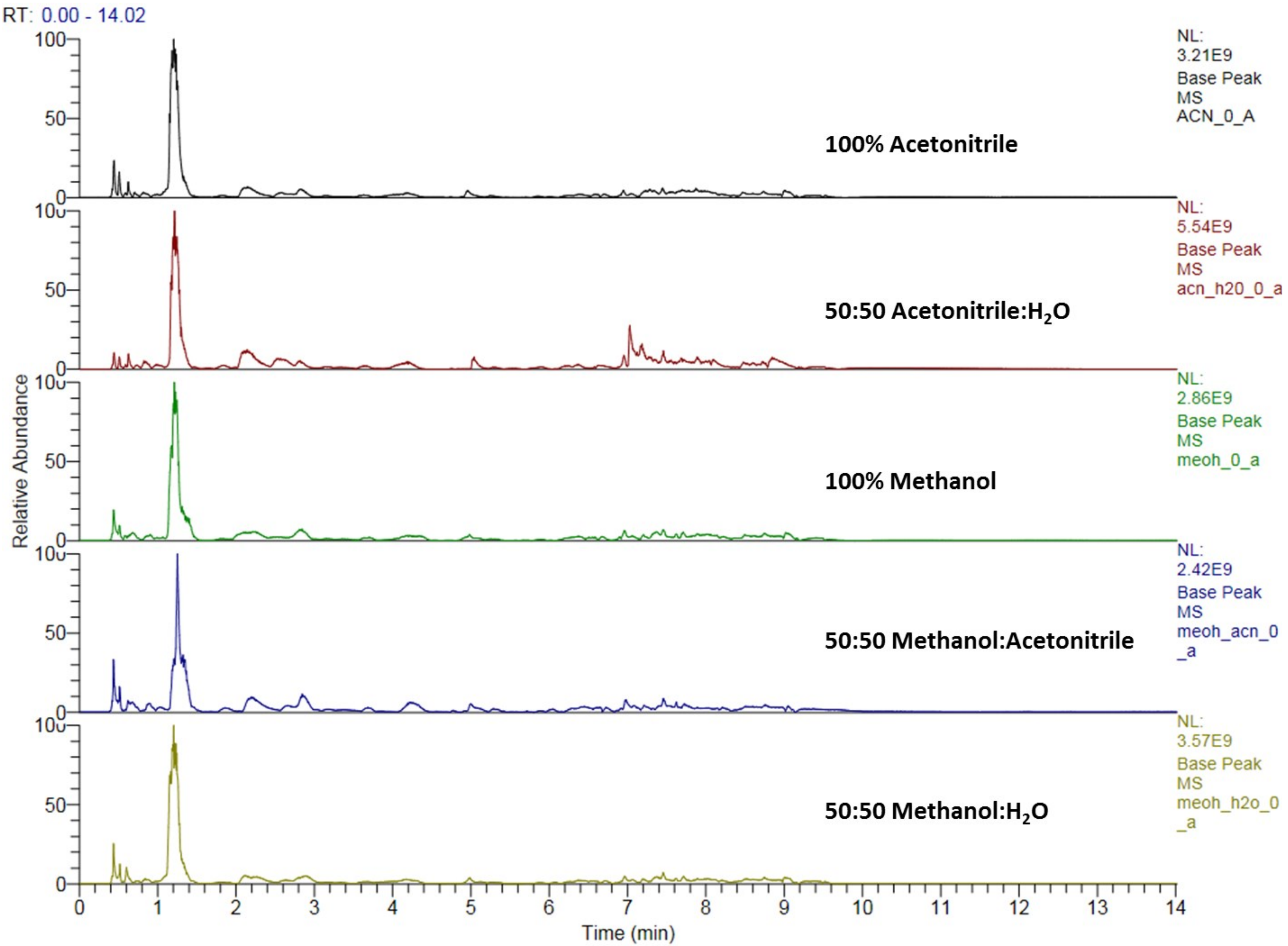

Figure S9 (B): Negative ion HILIC UHPLC-MS base peak chromatograms (mass range 70-1050 m/z) for the five monophasic urine extractions (with no solvent incubation).

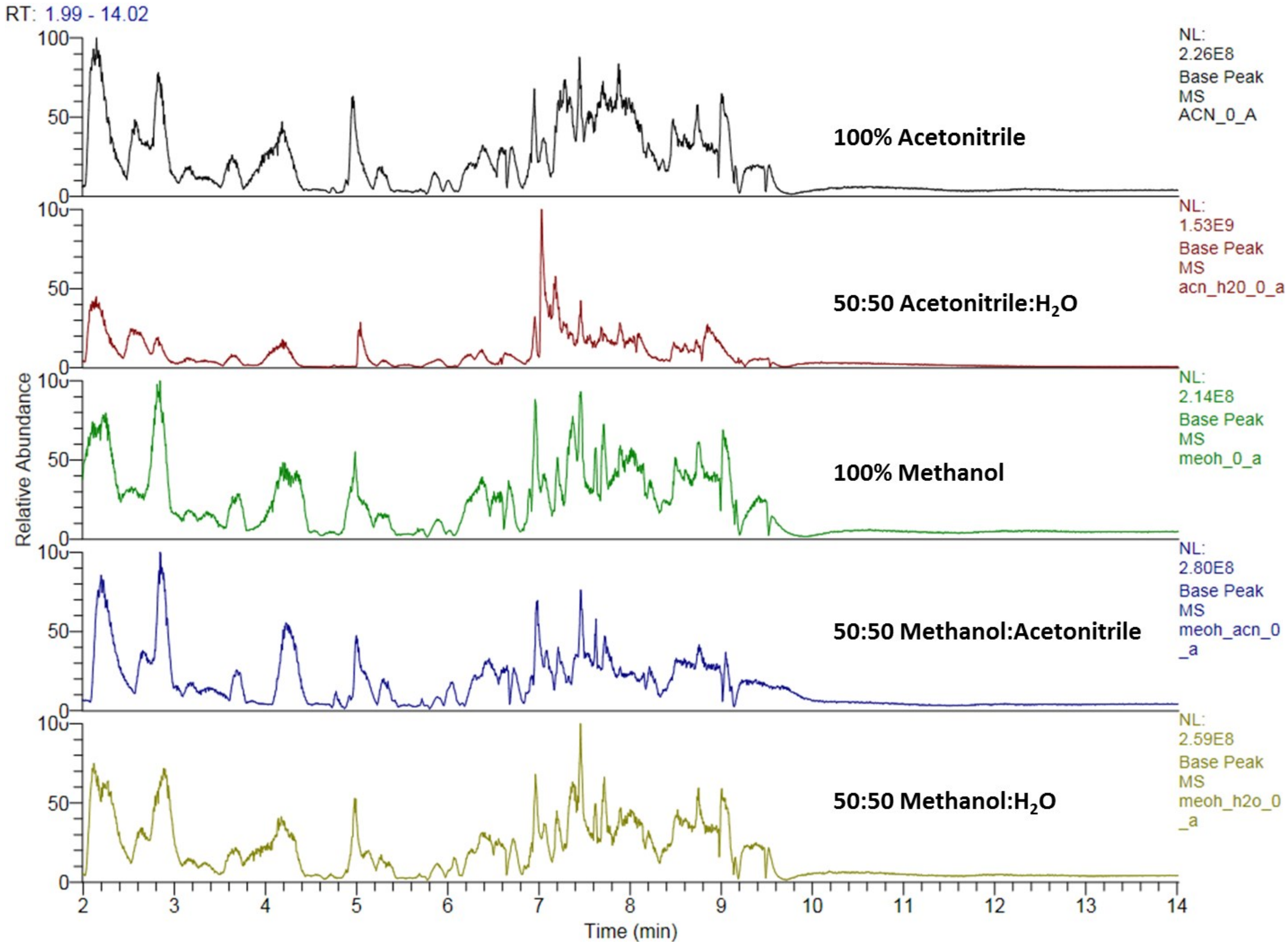

Figure S9 (C): Negative ion HILIC UHPLC-MS base peak chromatograms (mass range 70-1050 m/z) for the five monophasic urine extractions (with no solvent incubation). Zoomed on the retention time axis to show RT 2-14 min.

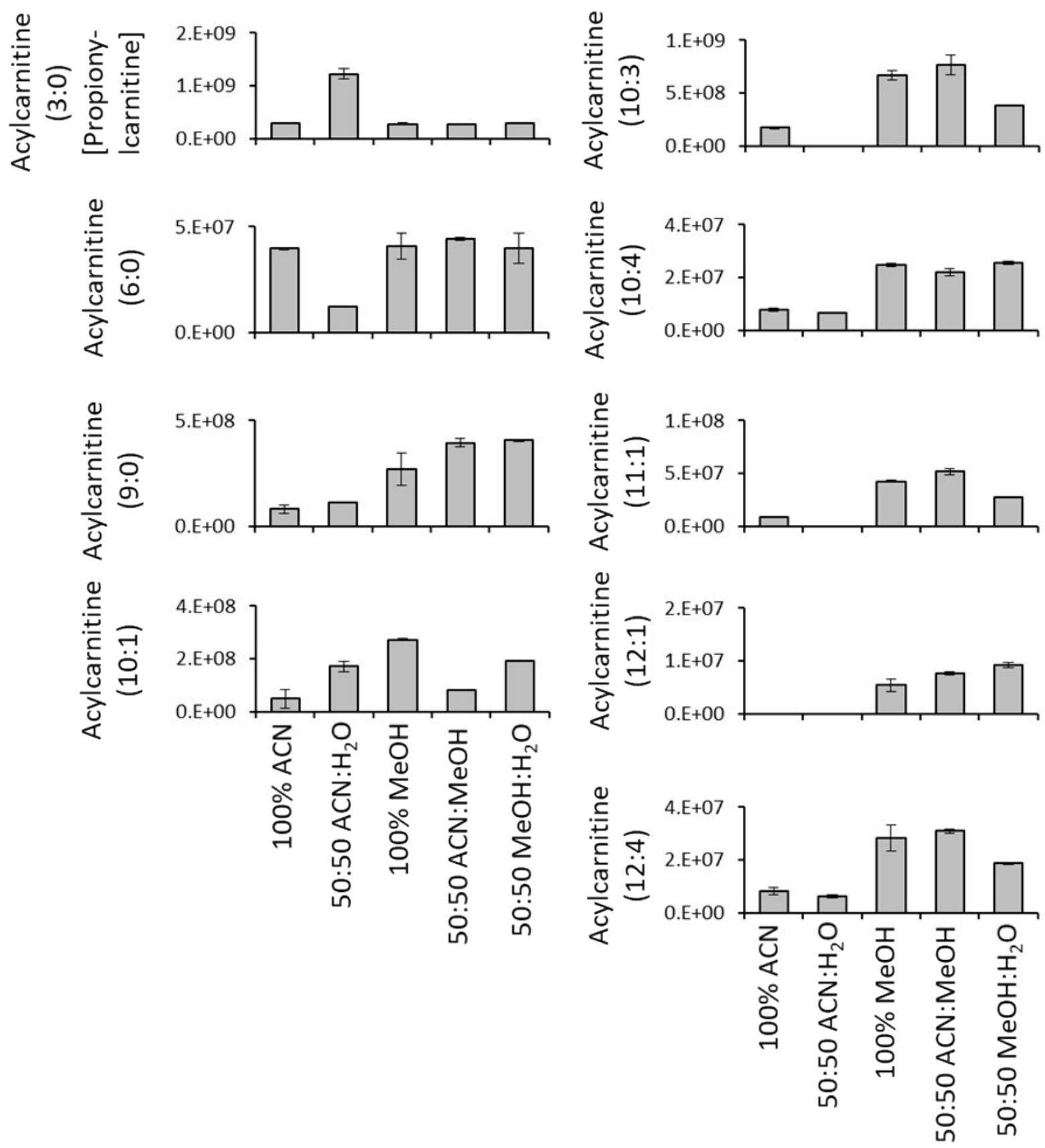

Figure S10: The detection responses (peak area) of individual acylcarnitine species (ionised as $[M+H]^+$ in positive ion mode) across all urine preparation methods by the UHPLC-MS HILIC assay in positive ion mode.

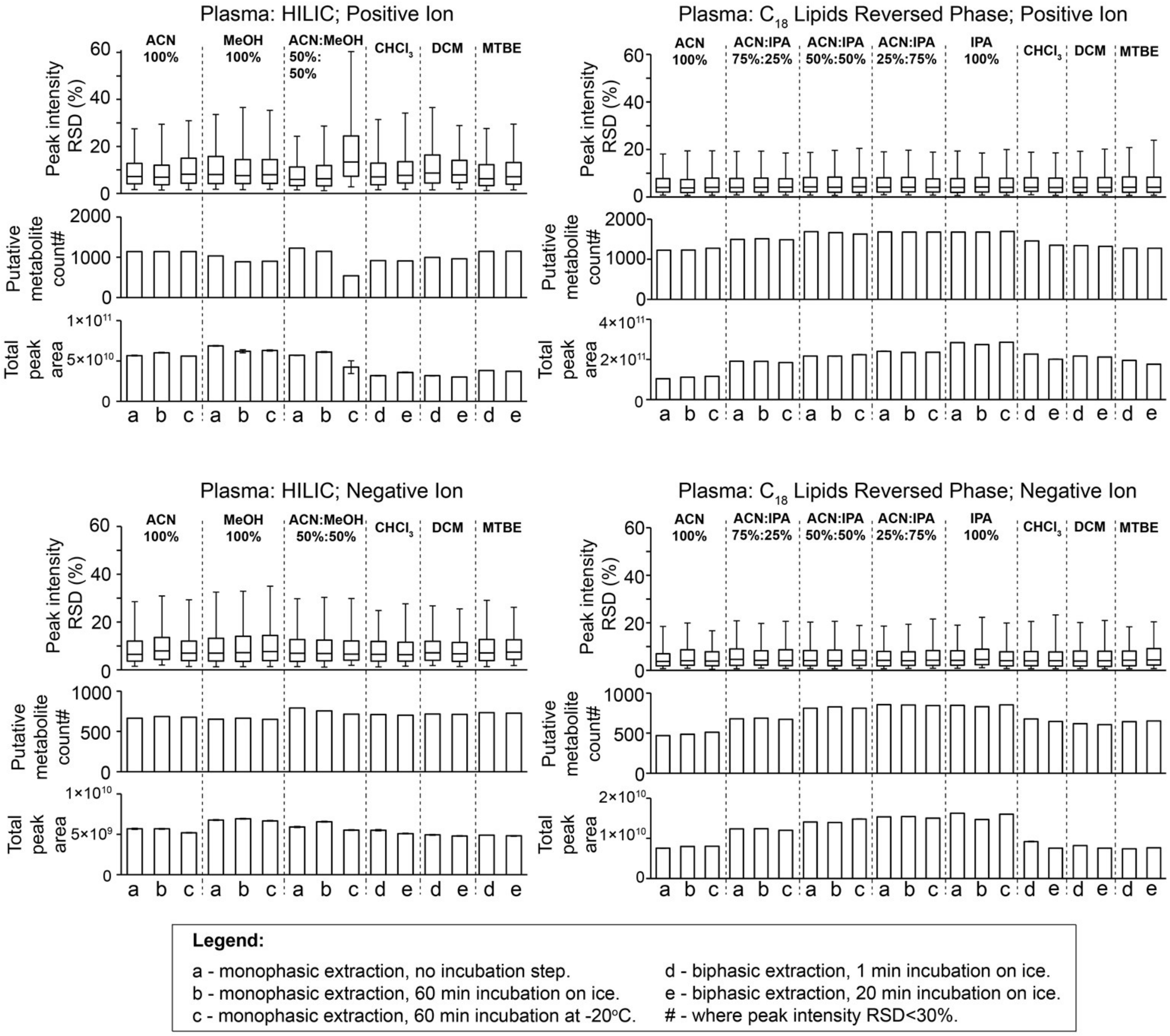

Figure S11: UHPLC-MS injection replicate data for putatively annotated metabolites from all plasma sample preparation methods. Data was filtered using QC samples and then putatively annotated (Experimental Section). Relative standard deviations (RSD; shown as interquartile ranges and error bars as the 95th percentile) are calculated on peak intensities where the putative metabolite is present in all three sample preparation replicates. Putatively annotated metabolite counts are those present in three sample preparation replicates with an intensity RSD<30%.

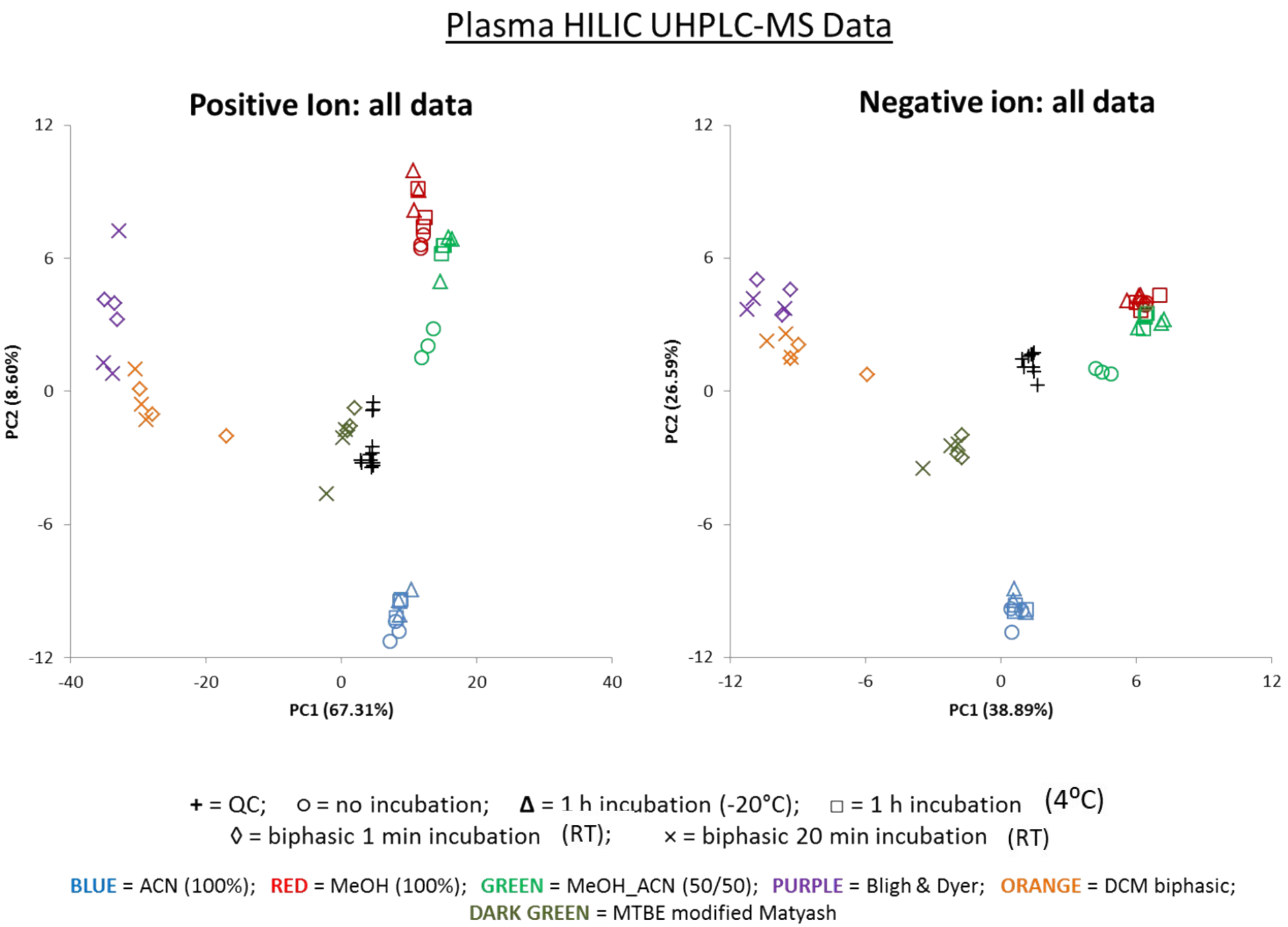

Figure S12: Principal components analysis (PCA) scores plots of the sample preparation replicates of each plasma extraction method analysed by HILIC UHPLC-MS analysis. RT = room temperature.

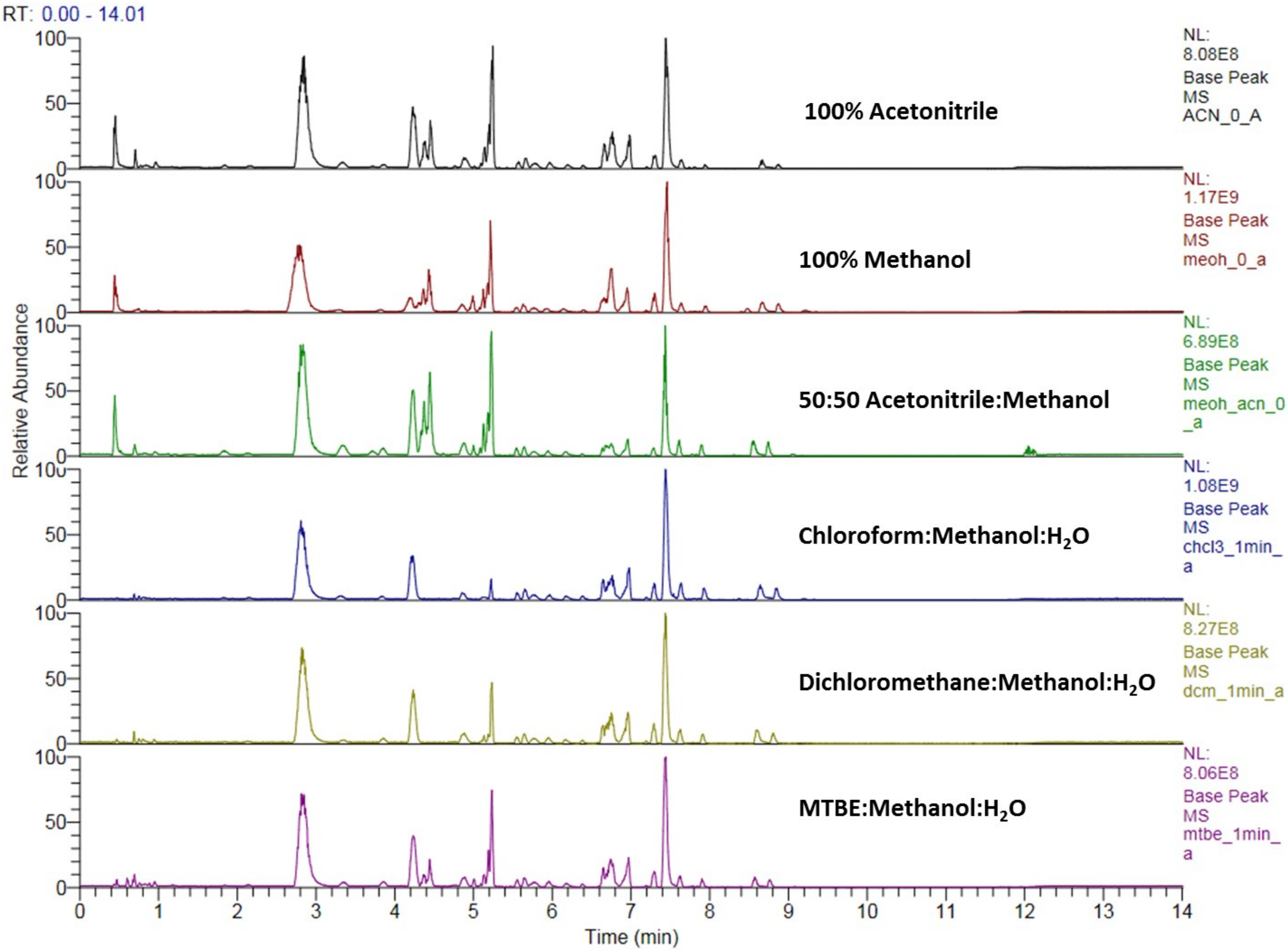

Figure S13 (A): Positive ion HILIC UHPLC-MS base peak chromatograms (mass range 70-1050 m/z) for the monophasic and biphasic plasma extractions (with no solvent incubation).

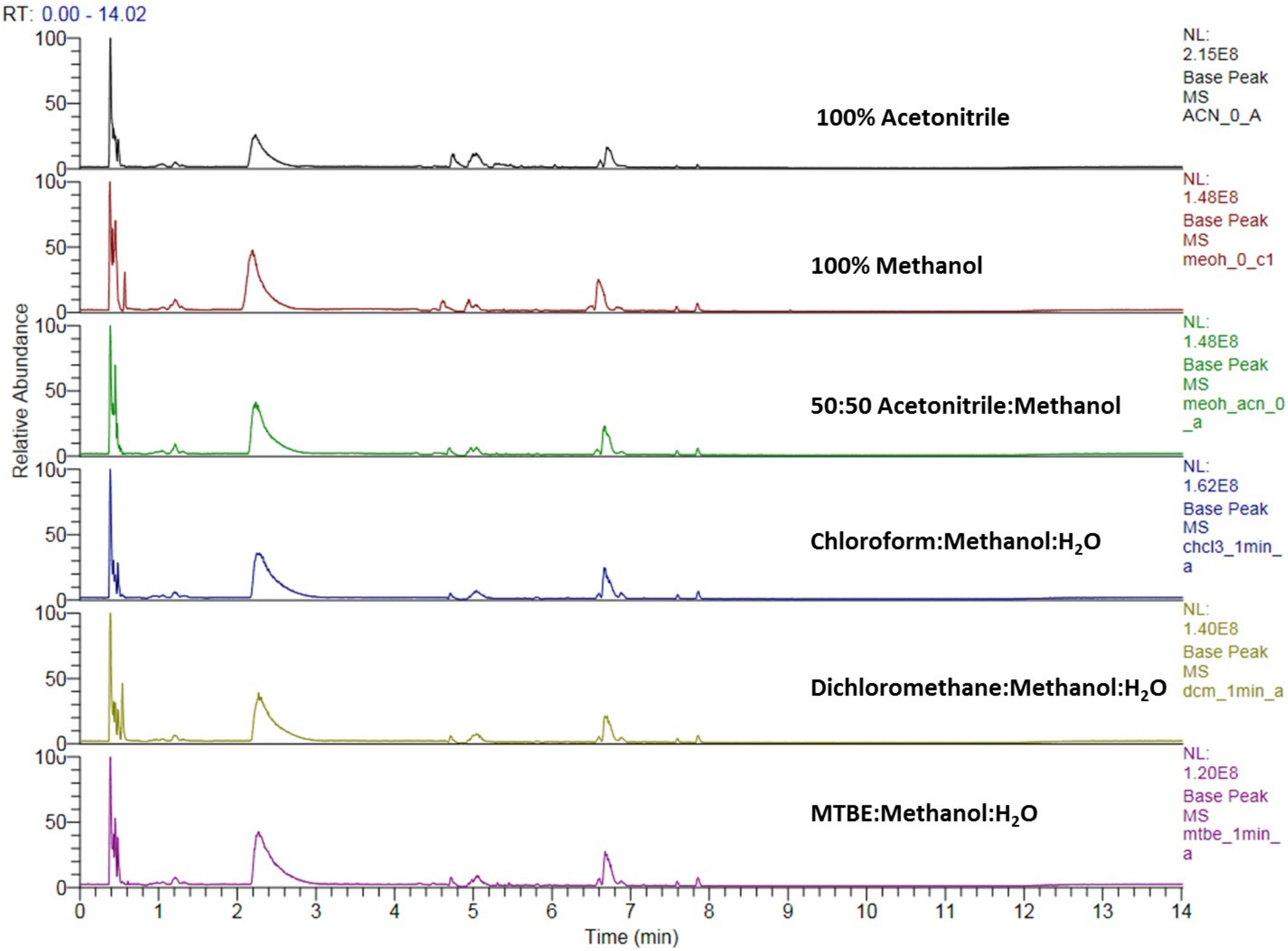

Figure S13 (B): Negative ion HILIC UHPLC-MS base peak chromatograms (mass range 70-1050 m/z) for the monophasic and biphasic plasma extractions (with no solvent incubation).

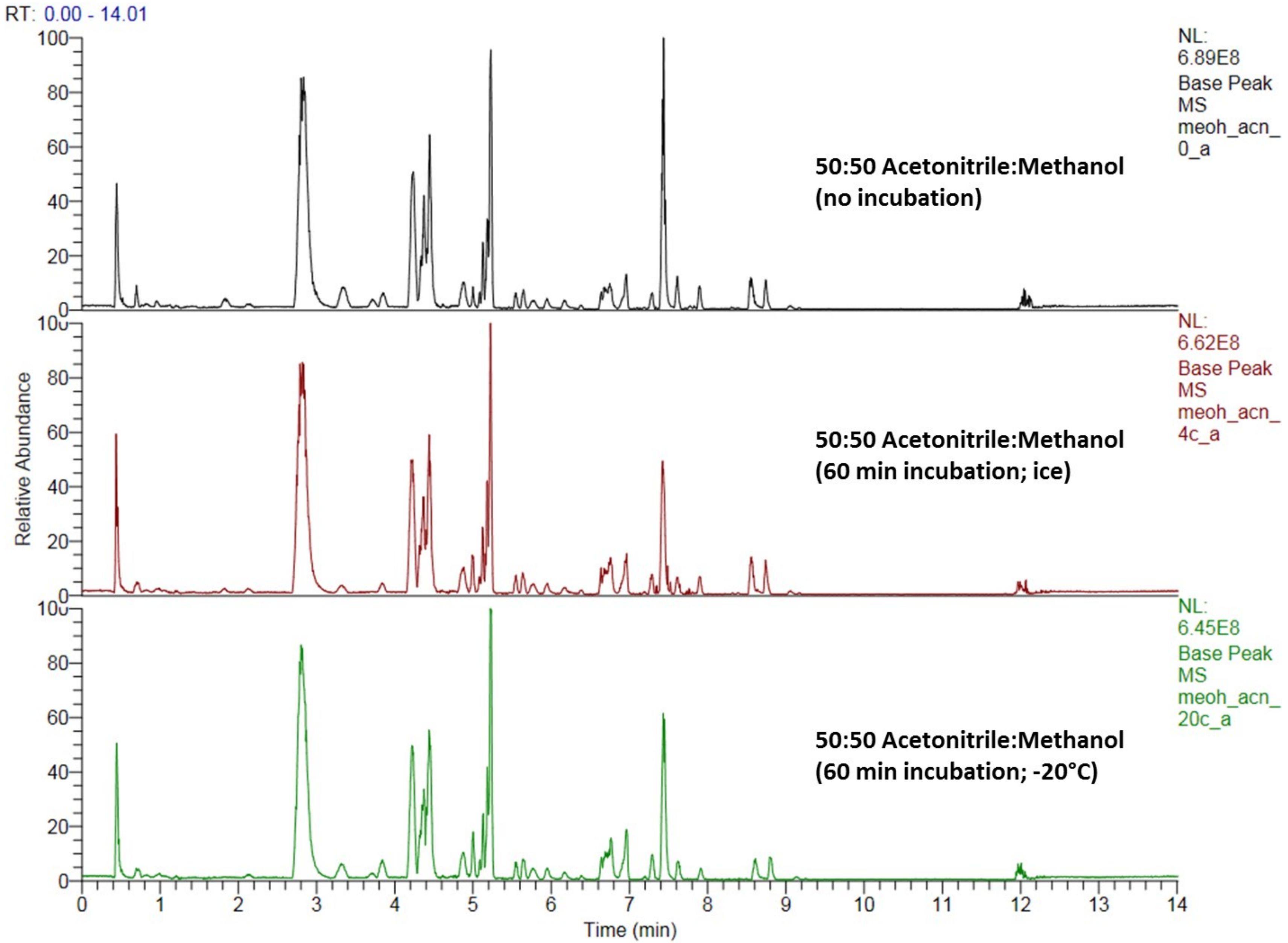

Figure S13 (C): Positive ion HILIC UHPLC-MS base peak chromatograms (mass range 70-1050 m/z) for the monophasic 50:50 ACN:MeOH extraction method with the different incubation options.

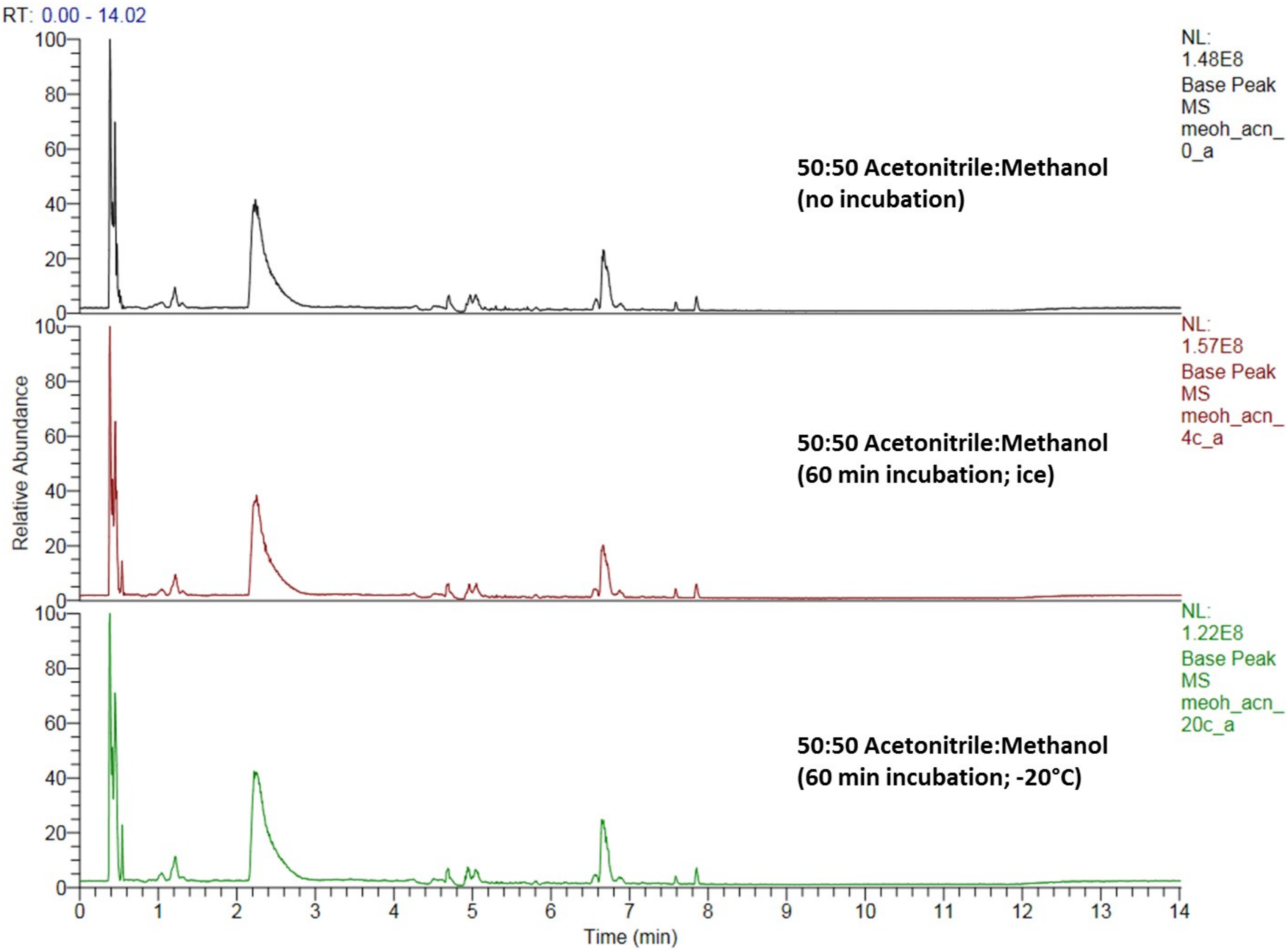

Figure S13 (D): Negative ion HILIC UHPLC-MS base peak chromatograms (mass range 70-1050 m/z) for the monophasic 50:50 ACN:MeOH extraction method with the different incubation options.

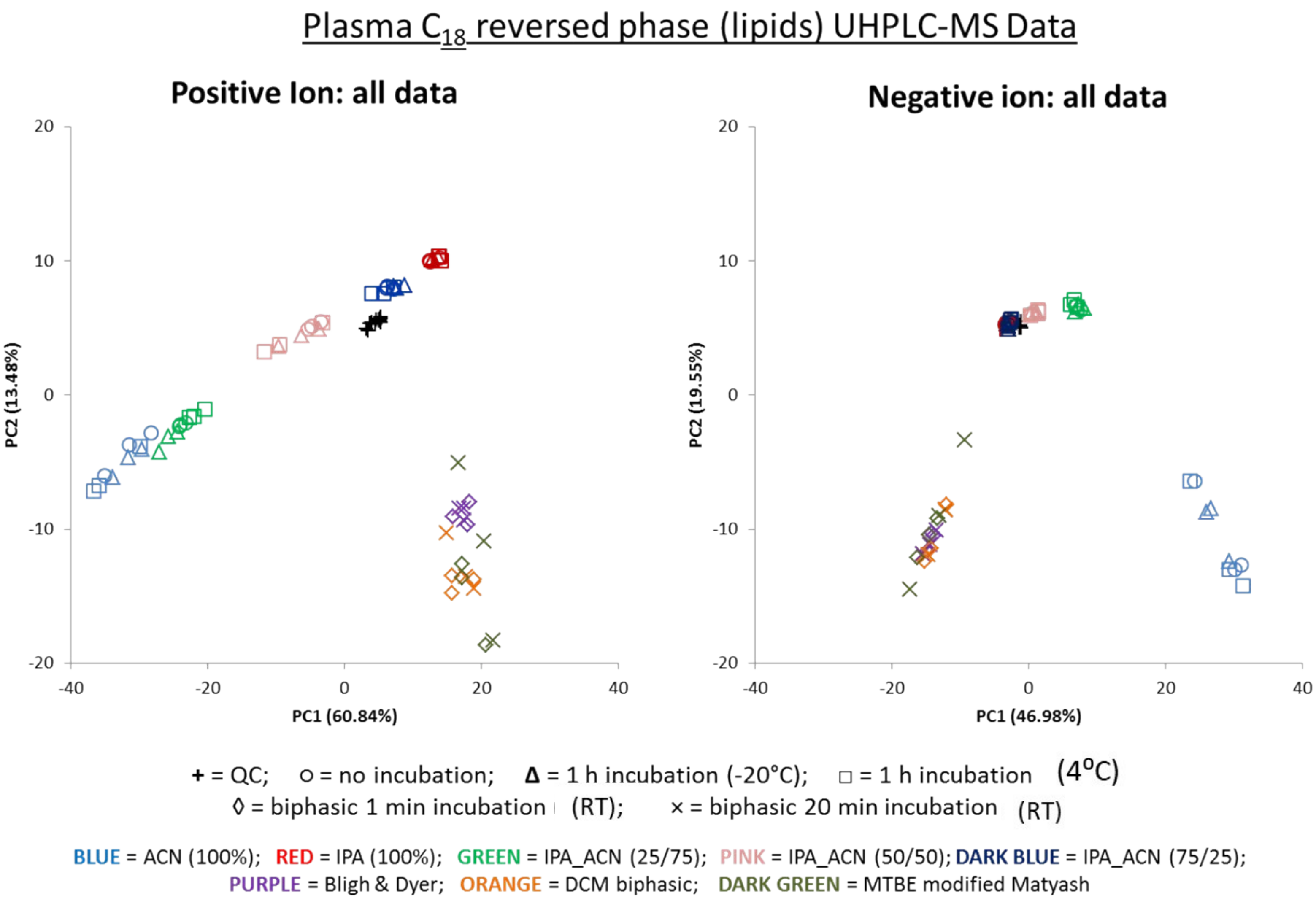

Figure S14: Principal components analysis (PCA) scores plots of the sample preparation replicates of each plasma lipid extraction method analysed by C_{18} UHPLC-MS analysis. RT = room temperature.

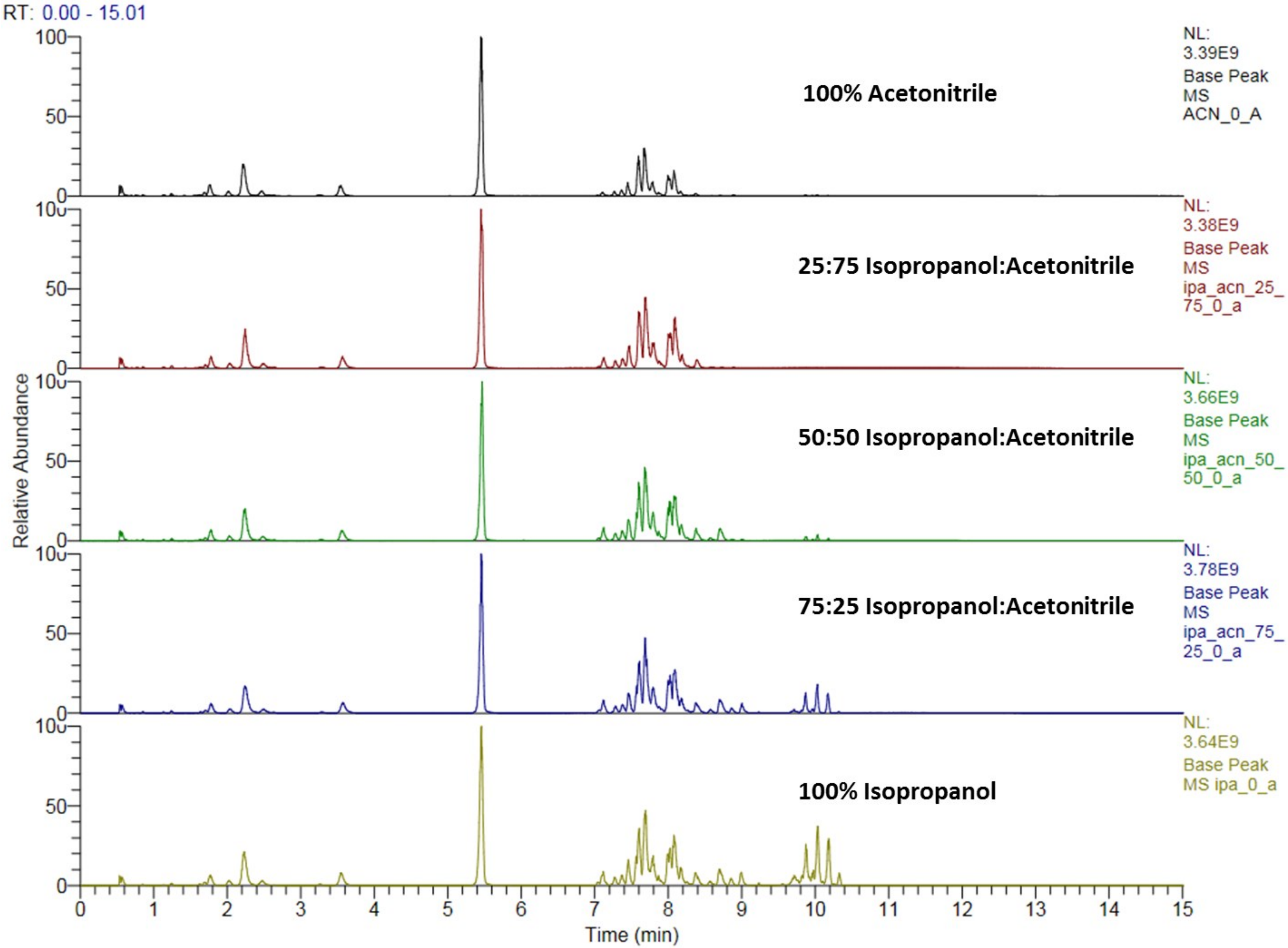

Figure S15 (A): Positive ion HILIC UHPLC-MS base peak chromatograms (mass range 150-2000 m/z) for the monophasic plasma lipid extractions (with no solvent incubation).

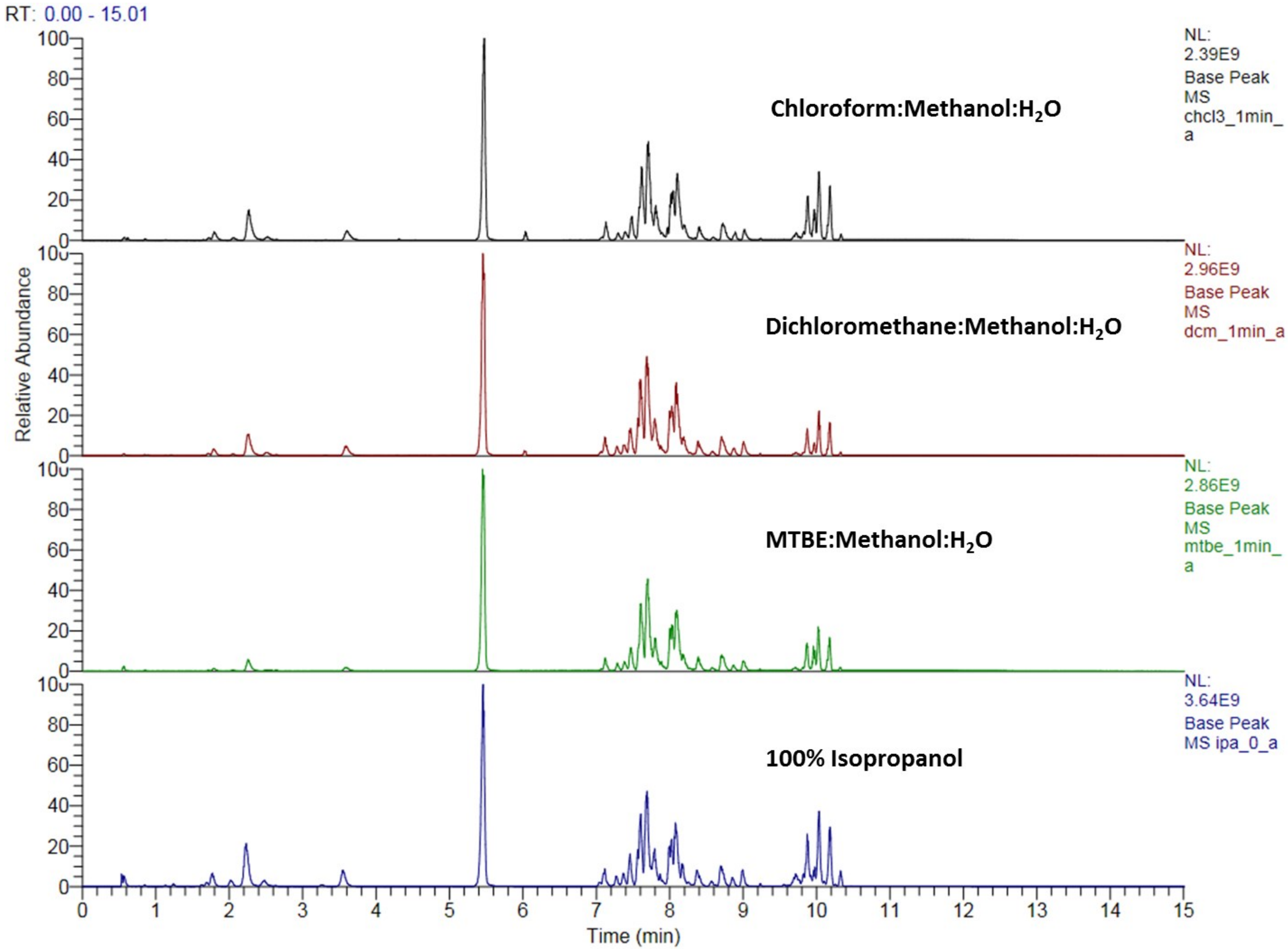

Figure S15 (B): Positive ion HILIC UHPLC-MS base peak chromatograms (mass range 150-2000 m/z) for biphasic plasma lipid extractions compared to the optimal 100% IPA monophasic extraction (with no solvent incubation).

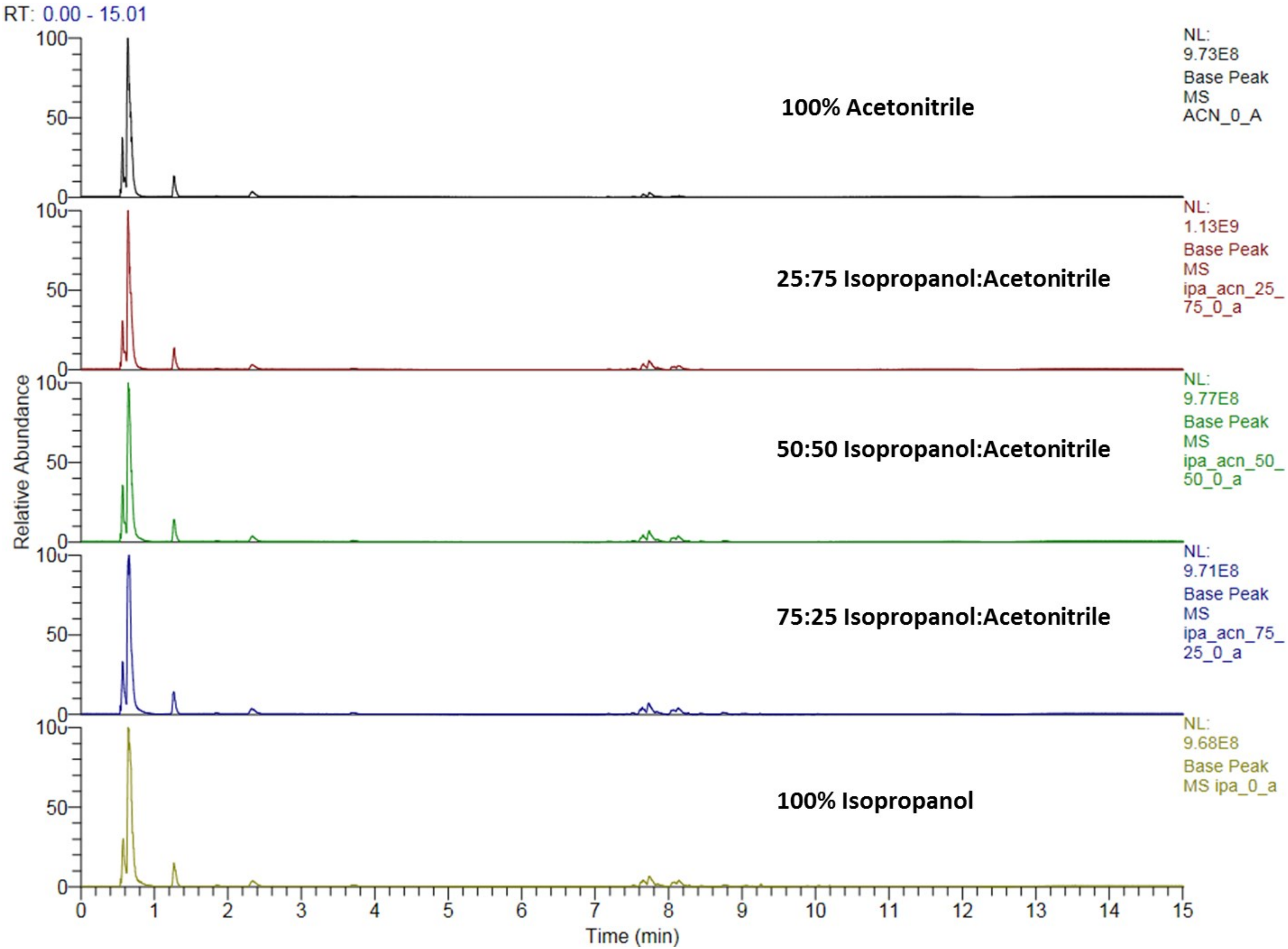

Figure S15 (C): Negative ion HILIC UHPLC-MS base peak chromatograms (mass range 150-2000 m/z) for the monophasic plasma lipid extractions (with no solvent incubation).

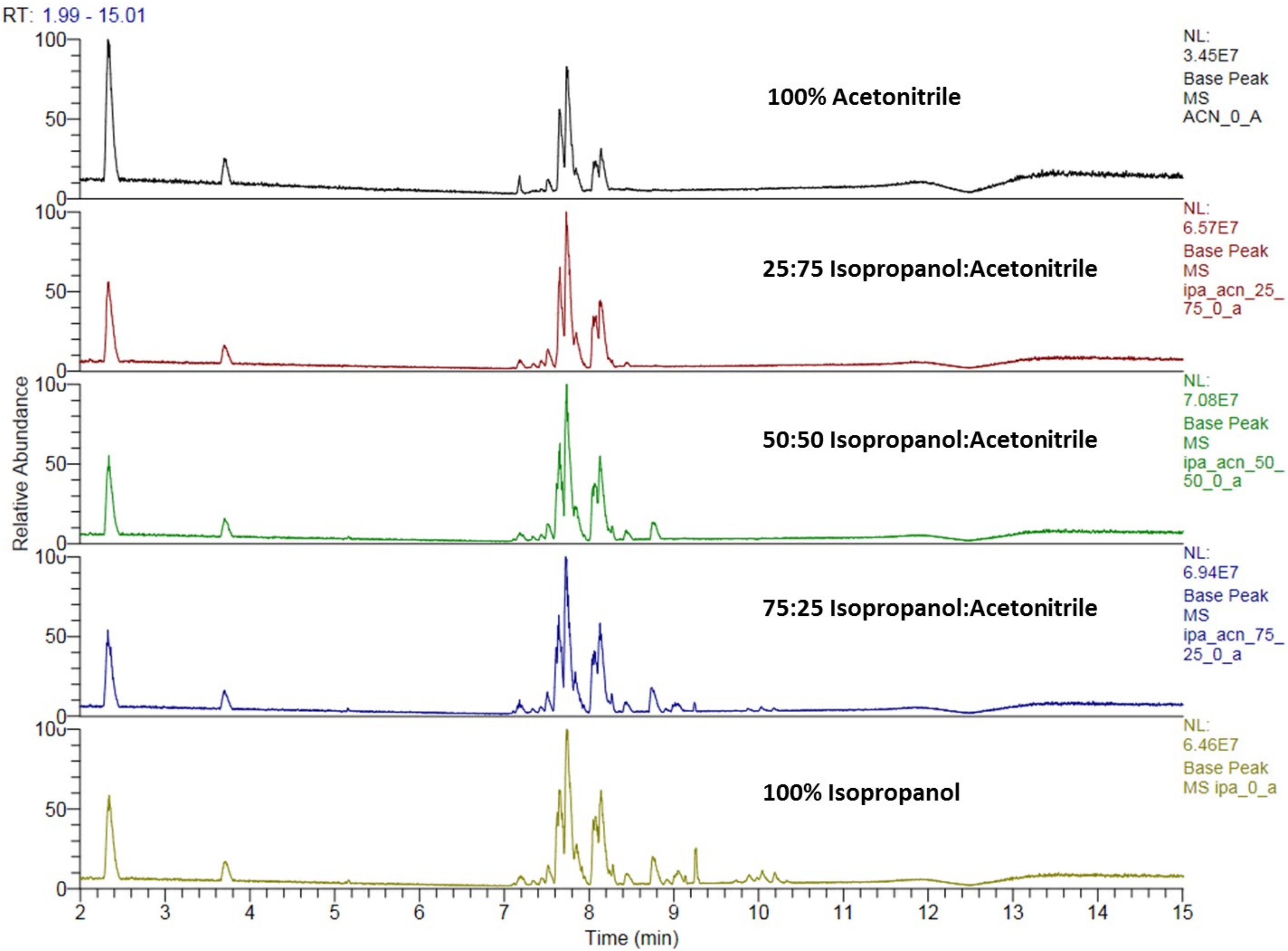

Figure S15 (D): Negative ion HILIC UHPLC-MS base peak chromatograms (mass range 150-2000 m/z) for the monophasic plasma lipid extractions (with no solvent incubation). Zoomed on the retention time axis to show RT 2-15 min.

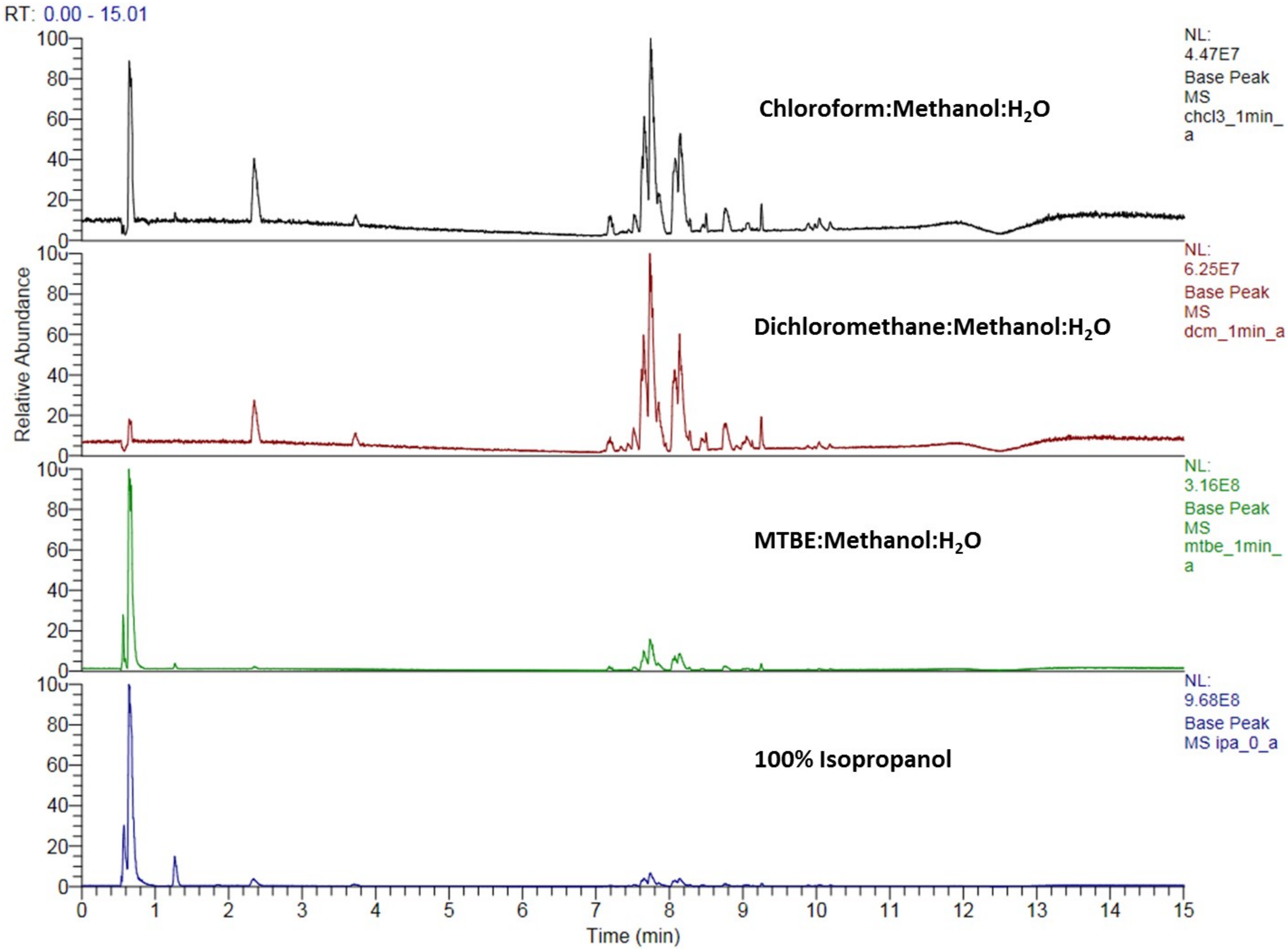

Figure S15 (E): Negative ion HILIC UHPLC-MS base peak chromatograms (mass range 150-2000 m/z) for biphasic plasma lipid extractions compared to the optimal 100% IPA monophasic extraction (with no solvent incubation).

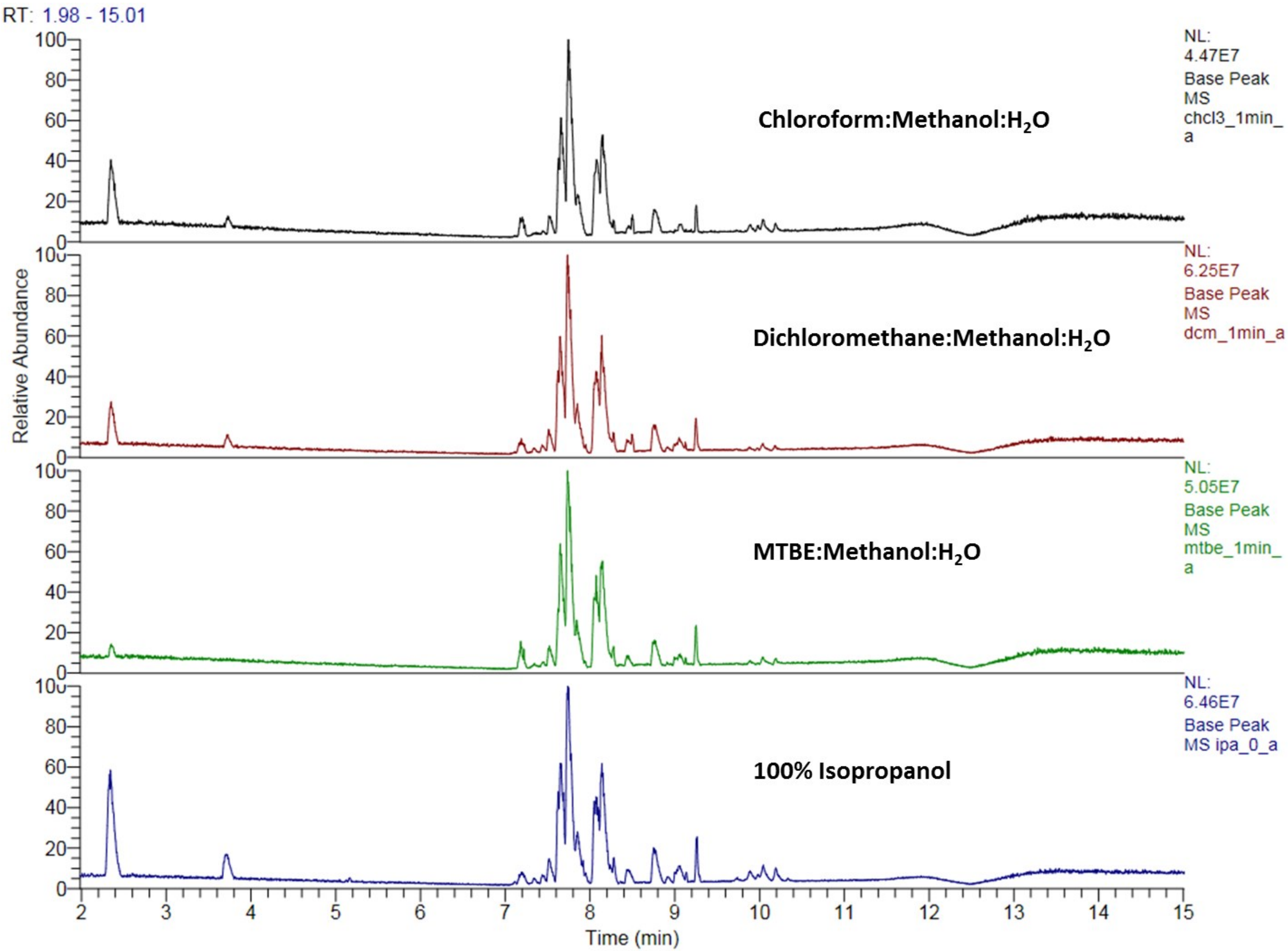

Figure S15 (F): Negative ion HILIC UHPLC-MS base peak chromatograms (mass range 150-2000 m/z) for biphasic plasma lipid extractions compared to the optimal 100% IPA monophasic extraction (with no solvent incubation). Zoomed on the retention time axis to show RT 2-15 min.
